# Leaf‐Inspired Eutectic Skin With Extreme Fatigue Resistance and Robust Wet Adhesion for Amphibious Epidermal Electronics

**DOI:** 10.1002/adma.73563

**Published:** 2026-05-29

**Authors:** Jiayu Hou, Jiancheng Dong, Je Hyeong Kim, Chang Zhou, Shiyin Lin, Xingyu Liu, Hao Qiu, Mengting Zheng, Yuduo Zhang, Haijun Zhu, Kangjia Geng, Yidong Peng, Haoran Liu, Yunpeng Huang, Yongsheng Luo, Steve Park, Tianxi Liu

**Affiliations:** ^1^ Key Laboratory of Synthetic and Biological Colloids Ministry of Education School of Chemical and Material Engineering Jiangnan University Wuxi China; ^2^ Department of Materials Science and Engineering Korea Advanced Institute of Science and Technology (KAIST) Daejeon Republic of Korea; ^3^ Kidney Transplantation Unit The First Affiliated Hospital of Zhengzhou University Zhengzhou China

**Keywords:** composite eutectogel, epidermal electronics, fatigue resistance, health monitoring, wet adhesion

## Abstract

Reliable skin‐interfacing electronics require soft materials that simultaneously tolerate repeated mechanical deformation and maintain robust adhesion in moist environments. However, conventional hydrogels are inherently limited by water‐induced swelling and interfacial failure. Inspired by the vein‐reinforced architecture of *Acorus calamus* leaves, we present a fatigue‐resistant and environmentally stable “eutectic skin” composed of an aligned polyurethane fibrous network embedded within a hydrophobic eutectogel matrix. The intrinsic hydrophobicity suppresses hydration to ensure exceptional dimensional stability with less than 1.1% swelling over 100 days. Crucially, the hierarchical fiber reinforcement imparts a unique “soft‐yet‐strong” mechanical behavior. The composite exhibits a tissue‐like softness (Shore A hardness of 13.6 A) yet achieves a true tensile strength of 106.51 MPa and a fatigue fracture threshold of 5.02 × 10^4^ J m^−^^2^ (≈3399‐fold enhancement) via strain‐induced crystallization. This exceptional toughness allows the material to sustain 100,000 cycles of notched stretching without crack propagation. The hydrophobic matrix also enables strong wet adhesion to skin (152.2 J m^−^^2^). This stable ionic interface supports high‐fidelity electrophysiological signal acquisition during underwater operation and continuous 7‐day monitoring. This work establishes a generalizable strategy for engineering mechanically resilient soft ionotronic interfaces for next‐generation wearable bioelectronics.

## Graphical abstract

Inspired by Acorus calamus leaves, a heterogeneous eutectic skin integrates an aligned fibrous network within a hydrophobic eutectogel matrix. This hierarchical architecture triggers strain‐induced crystallization to achieve exceptional mechanical toughness and durable wet adhesion. The anti‐swelling composite provides a highly robust soft ionotronic interface for reliable and continuous epidermal electrophysiological monitoring under demanding environmental conditions.

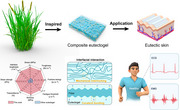

## 1 Introduction

Skin electronics have revolutionized emerging fields such as smart wearables, continuous health monitoring, and humanoid robotics by virtue of their tissue‐like softness and ability to form intimate, conformal contact with diverse surfaces [1, 2, 3, 4, 5, 6, 7, 8, 9, 10]. In particular, ionically conductive gels (e.g., hydrogels) are promising materials for serving as critical interfaces that bridge electronic devices and biological tissues [11, 12, 13]. However, conventional hydrogels suffer from inevitable dehydration in open air and swelling in wet environments. These instabilities lead to the leakage of conductive media and a dramatic decline in sensing performance [14]. While organogels and ionogels offer non‐volatile alternatives, they are often limited by potential skin irritation and biotoxicity [15].

In this context, hydrophobic deep eutectic solvents (HESs) have attracted significant attention for their biocompatibility, low volatility, and ionic conductivity [16]. Eutectogels derived from HESs preserve these advantages and possess a stable hydrogen‐bond network [17]. Despite their potential, most reported eutectogels remain mechanically weak and brittle, with tensile strength often limited to 10–100 kPa and fracture energies on the order of 10–1000 J m^−^^2^ [18, 19, 20, 21, 22, 23]. As a consequence, repeated static and dynamic cyclic loading of eutectogels under long‐term and substantial deformation will cause localized relaxation and slip of polymer macromolecular chains in gel matrix, leading to crack initiation, and eutectogel fatigue failure [24, 25]. Enhancing the mechanical robustness and fatigue resistance of eutectogels is thus a central challenge that must be overcome to realize durable skin‐interfacing electronics [26, 27].

To address mechanical fragility, toughening strategies such as double‐network structuring [28], freeze thawing induced crystallization [29], and hierarchical design have been proposed [30]. Among them, poly(vinyl alcohol) (PVA)‐based eutectogels are most widely studied due to the dense hydrogen bonding provided by their abundant hydroxyl groups [23, 31, 32, 33, 34]. However, PVA eutectogels are inherently hydrophilic and readily swell in humid environments, which weakens intermolecular interactions, degrades mechanical performance, and compromises both conductivity and long‐term stability. In contrast, fiber reinforcement provides a universal, and highly effective strategy for strengthening gel matrices [35, 36, 37, 38]. Incorporating a tough fibrous mesh into a hydrophobic eutectogel matrix enables efficient stress redistribution and energy dissipation during cyclic loading while preserving interfacial integrity and electrochemical stability under wet conditions. Nevertheless, achieving durable wet adhesion remains a major challenge for epidermal electronics, as the low surface energy (25–29 mN·m^−^^1^) and intricate microtexture of human skin hinder most eutectogels from maintaining stable contact during perspiration, motion, or prolonged wear [39].

Herein, a fatigue‐resistant and wet‐adhesive composite eutectogel, termed the eutectic skin, was rationally designed, drawing structural inspiration from the robust leaf architecture of *Acorus calamus* (sweet flag). We fabricated a surface‐functionalized aligned polyurethane (PU) fiber mesh to mimic the reinforcing leaf veins and infiltrated it with a hydrophobic eutectogel precursor. The subsequent in situ polymerization creates a hierarchical interface where covalent linkages, hydrogen bonding, and microscale mechanical interlocking ensure efficient stress transfer. This design imparts exceptional fatigue tolerance and prevents delamination under dynamic deformation. Furthermore, the incorporation of specific hydrophobic monomers enhances the cohesive energy of the matrix and promotes mechanical anchoring to skin microtextures. The resulting eutectic skin integrates biocompatibility, hydrophobicity, strong interfacial coupling, and exceptional mechanical resilience. We demonstrate its capability as a versatile bioelectrode for high‐fidelity electrophysiological signal acquisition and reliable long‐term health monitoring in realistic environments. This versatile and durable platform represents a significant step forward for next‐generation epidermal electronics.

## 2 Results and Discussion

### 2.1 Design of Robust Leaf‐Inspired Eutectic Skin

**FIGURE 1:**
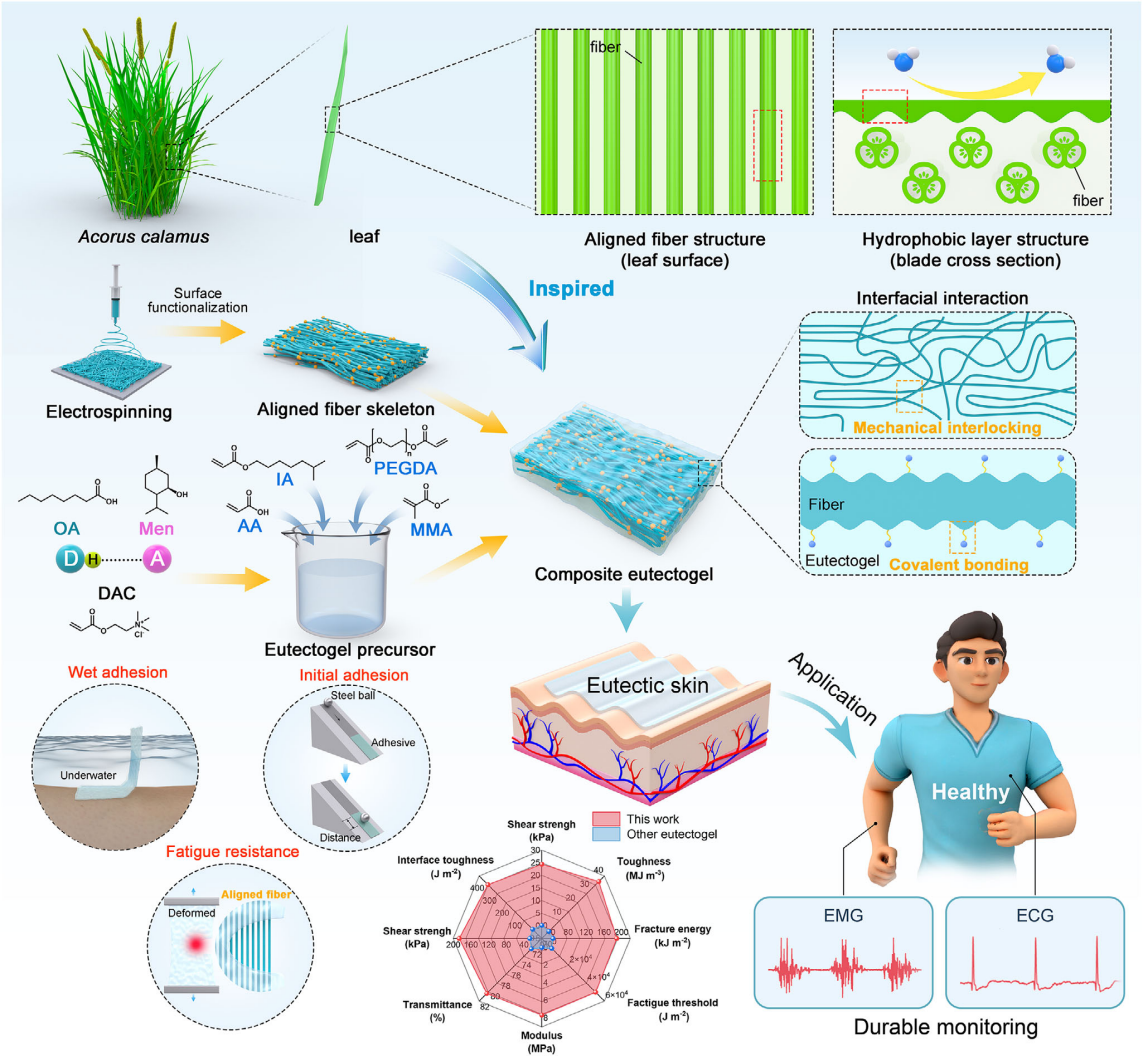
Display of *Acorus Calamus* leaf inspired composite eutectogel with superior comprehensive performance for durable amphibious epidermal electronics.

*Acorus calamus* is a wetland plant whose leaves are naturally adapted to withstand continuous water flow and strong winds while maintaining efficient water storage and transport. Unlike terrestrial plants such as maize and sorghum, *Acorus calamus* lacks a petiole, causing its leaves to directly bear external mechanical loads and thereby develop exceptional bending tolerance and resistance to fracture [40, 41]. In addition, volatile oils secreted by specialized oil cells reduce the surface energy of the fibrous matrix, effectively inhibiting water infiltration. Meanwhile, the internal fibrous network is organized in a hierarchical architecture that synergistically combines mechanical stability with efficient mass transport and diffusion (Figure 1 and Figure S1) [42, 43].

Motivated by this biological strategy of integrating internal mechanical stability with external hydrophobic protection, we developed a eutectic skin. A composite eutectic skin was fabricated by integrating an aligned PU fibrous mesh with a hydrophobic eutectogel matrix. The electrospun PU fibers function as a reinforcing skeleton analogous to the fibrous framework of the leaf, while the eutectogel matrix provides stable ionic conduction within a water‐repellent environment, mimicking the protective yet functional surface of the leaf.

The chemical composition of the eutectogel matrix was specifically engineered to optimize both internal cohesion and interfacial interaction. Isooctyl acrylate (IA), acrylic acid (AA), and methyl methacrylate (MMA) were selected as the primary monomers. The long‐chain alkyl side chains of IA segments enable the formation of a mechanical interlocking structure with various substrate surfaces. Acrylic acid was incorporated to leverage its carboxyl groups (─COOH) which significantly enhance hydrogen bonding interactions between the polymer network and the HES components. Concurrently, MMA segments effectively increase the cohesive energy of the gel to prevent cohesive failure during detachment. The ternary HES itself provides the necessary ionic conductivity while maintaining the intrinsic hydrophobicity required for environmental stability (Figure 1).

This structural integration offers two complementary advantages. First, the aligned fibrous network redistributes mechanical stress and suppresses crack initiation and propagation under cyclic deformation. Second, the eutectogel matrix forms robust interfacial interactions with the underlying substrate, enabling stable adhesion even under humid conditions. Together, these features yield a compliant, silicone‐free interface that is resistant to mechanical fatigue while maintaining reliable wet adhesion and stable electrochemical performance, all of which are critical for high‐performance skin electronics [44]. As a result, the composite eutectic skin simultaneously exhibits mechanical resilience, fatigue resistance, optical transparency, and durable wet adhesion‐an integrated set of properties that is difficult to achieve with previously reported eutectogel systems. Such multifunctional performance positions this platform as a robust and versatile interface for skin electronics and long‐term health‐monitoring applications [33, 45, 46, 47, 48, 49, 50].

### 2.2 Morphological, Optical, and Inherent Anti‐Swelling Properties

**FIGURE 2:**
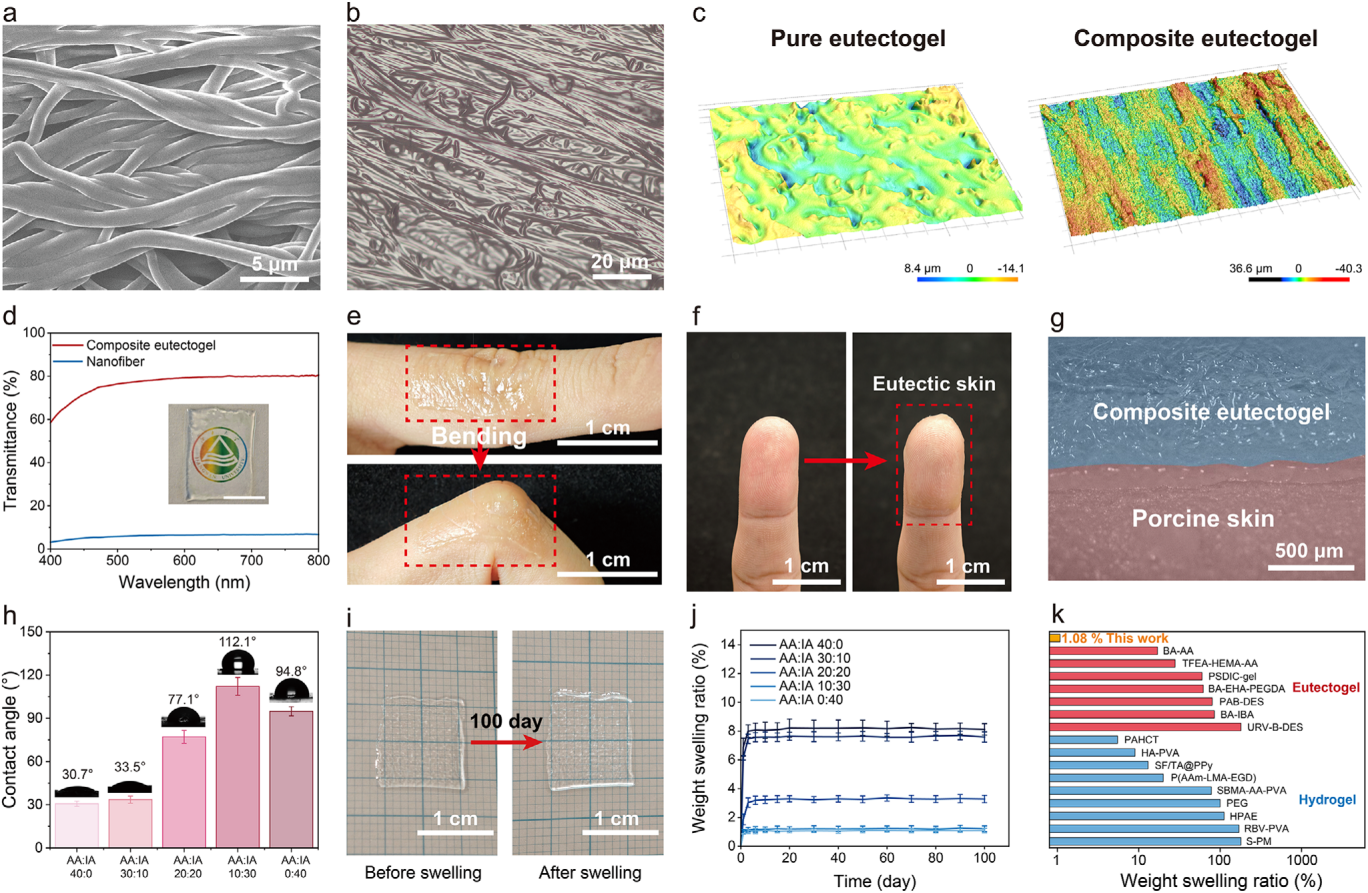
Structure and hydrophobicity of composite eutectogel. (a) SEM image of aligned PU fiber skeleton. (b) Optical microscope image of composite eutectogel. (c) Laser microscope images of pure eutectogel and composite eutectogel. (d) Transmittance of composite eutectogel and fiber skeleton. Demonstration of conformal fitting: (e) eutectogel attached on finger joint and (f) finger tip. (g) Optical microscope images of the adhesion between composite eutectogel and porcine skin interface. (h) Contact angles with different eutectogel compositions. (i) Digital photographs showing composite eutectogel prior to and after swelling in water over 100 days. (j) Weight swelling ratio with different compositions. (k) Comparison of weight swelling ratio with recently reported anti‐swelling eutectogels and hydrogels.

The morphology of the aligned PU fibrous mesh and the composite eutectogel is shown in Figure 2a,b. The fibrous mesh exhibits a high degree of alignment with a narrow fiber diameter distribution (mean diameter: 0.49 ± 0.16 µm; Figures S2 and S3). Upon being impregnated with precursors followed by in situ polymerization, the aligned PU mesh was merged with the eutectogel to construct a unified hybrid mesh (Figure 2c and Figures S4 and S5). Notably, the fibers retain their aligned configuration within the eutectogel framework, resulting in an anisotropic architecture reminiscent of the aligned fibrous structure observed in *Acorus calamus* leaves. In addition, modifications to the fiber surface microstructure create abundant interfacial sites that promote mechanical interlocking between the eutectogel matrix and the fibrous reinforcement.

The composite eutectogel exhibits high optical transmittance in the visible region (Figure 2d). In contrast, the pristine PU fibrous mesh shows a low transmittance of only 6.7%, whereas the fiber‐reinforced eutectogel achieves transmittances of 78.1% at 550 nm‐corresponding to the peak sensitivity of the human eye and 80.5% at 800 nm [51]. This enhancement arises from refractive‐index matching between the eutectogel matrix and the PU fibers, as well as the elimination of air gaps within the fibrous network, which collectively reduce refractive‐index contrast and light scattering [52]. Furthermore, the transparent eutectic skin exhibits excellent surface compliance, enabling it to conform intimately to complex epidermal geometries such as fingerprints and finger joints (Figure 2e,f). This ultraconformal contact facilitates stable and reliable acquisition of electrophysiological signals. Cross‐sectional optical microscopy reveals continuous, gap‐free contact between the eutectic skin and a skin replica (Figure 2g), confirming strong interfacial coupling that is essential for reproducible signal transduction.

In addition, the eutectogel exhibits a high intrinsic level of hydrophobicity, as evidenced by the increase in water contact angle with increasing IA content (Figure 2h). Specifically, the water contact angle increases from 30.7° to 112.1° upon tuning the composition of the polymerized monomers (Table S1). This trend reflects the enhanced hydrophobicity imparted by the long alkyl side chains of IA [53]. Meanwhile, the introduction of a small fraction of AA further optimizes the polymer network through hydrogen‐bond‐mediated chain organization, contributing to improved hydrophobic characteristics. Consistent with this behavior, swelling tests reveal that increased hydrophobicity leads to a substantial reduction in the equilibrium swelling ratio, decreasing from 8.1% to 1.1%. Notably, no measurable swelling is observed even after 100 days of continuous immersion, demonstrating the exceptional dimensional stability of the eutectogel under prolonged wet conditions (Figure 2i,j).

Compared with recently reported anti‐swelling eutectogels and hydrogels, our composite exhibits markedly reduced swelling (Figure 2k and Table S2). This suppressed swelling is particularly critical for fiber‐reinforced eutectogels, as it minimizes volumetric expansion and alleviates interfacial stress between the eutectogel matrix and the fibrous mesh [54, 55, 56, 57]. For epidermal electronics, the combined hydrophobicity and low swelling enhance moisture resistance and long‐term durability, mitigating performance degradation caused by water uptake while preserving stable electrochemical and mechanical behavior under humid and dynamic conditions [58].

### 2.3 Mechanical Performance

**FIGURE 3:**
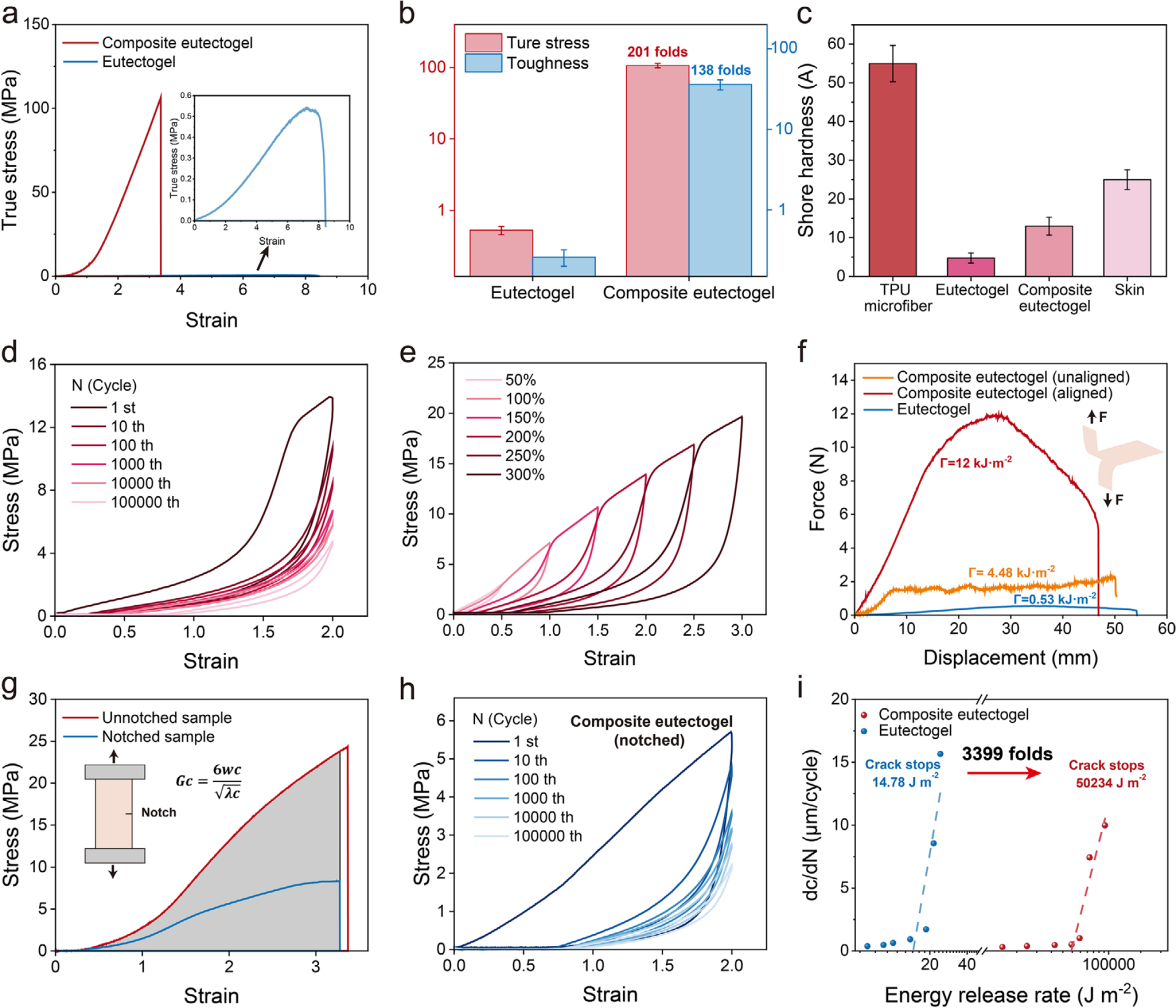
Mechanical performance of composite eutectogel. True stress‐strain curves (a) alongside values comparison (b) of composite eutectogel and pure eutectogel (inset in Figure 3a depicts pure gel). (c) Shore hardness of TPU microfiber, pure eutectogel, composite eutectogel, and human skin. (d) Cyclic curves of composite eutectogel after 100 000 stretching cycles. (e) Cyclic curves of composite eutectogel after 50–300% elongation. (f) Trouser tear curves, and (g) notch tensile test of composite eutectogels. (h) Cyclic curves of notched composite eutectogel after 100 000 stretching cycles. (i) Fatigue threshold of composite eutectogel and pure eutectogel.

The mechanical properties of the composite eutectogels were systematically characterized (Figure 3). Compared with the pure eutectogel and the unaligned composite, the aligned fiber‐reinforced eutectogel exhibits a dramatic enhancement in both tensile strength and toughness. Even in the perpendicular direction, its mechanical performance remains superior to that of the pure eutectogel (Figure S6). Specifically, the true tensile strength increases from 0.53 to 106.51 MPa, corresponding to an approximately 201‐fold enhancement, while the toughness rises from 0.26 to 35.98 MJ m^−^^3^ (≈138‐fold increase), as shown in Figure 3a,b. Despite the substantial increases in strength and toughness, the composite retains skin‐like softness, exhibiting a Shore A hardness of 13.6 A, which is comparable to that of human skin (≈25.5 A), as shown in Figure 3c. This unusual combination of high mechanical robustness and mechanical compliance enables the material to remain comfortable while providing sufficient durability, highlighting its strong potential for wearable electronic applications.

The aligned fiber‐reinforced eutectogel exhibits excellent cyclic stability and tear resistance. Notably, the composite maintains similar stress–strain responses after 100 000 loading cycles (Figure 3d), indicating efficient energy dissipation mediated by the aligned fiber network. In addition, the composite eutectogel was subjected to progressive cyclic strain sweeps with increasing maximum strains from 50% to 300% (Figure 3e). Across all strain levels, the stress–strain curves remain smooth and continuous, with no abrupt stress drops or mechanical instability. These results indicate that the composite eutectogel can withstand repeated stretching to strains of up to 300% without permanent mechanical degradation, even under deformation amplitudes representative of extreme daily activities. Moreover, the aligned fiber‐reinforced eutectogel exhibits a high tear energy of 12 kJ m^−^^2^ with no observable notch propagation during stretching (Figure 3f). In contrast, the randomly aligned fiber composite and the pure eutectogel show much lower tear energies of 4.48 and 0.53 kJ m^−^^2^, respectively. These results demonstrate that fiber alignment not only enhances mechanical strength but also significantly improves resistance to crack propagation, a critical attribute for the durability and reliability of wearable electronic devices.

The fracture energy of the fiber‐reinforced eutectogel was quantified using unilateral notch tensile tests. As shown in Figure 3g, the notched composite sustains strains up to 327%, comparable to the unnotched sample (337%), with a high fracture energy of 189.3 kJ m^−^^2^. Because fracture energy under monotonic loading does not necessarily correlate with fatigue resistance under cyclic loading [59], cyclic tensile tests were performed on notched composite and pure eutectogel samples. After 100 000 cycles at a fixed strain of 200%, the composite exhibits negligible crack propagation (Figure 3h). The corresponding fatigue fracture threshold is ∼5.02 × 10^4^ J m^−^^2^, approximately 3,399 times higher than that of the pure eutectogel (14.78 J m^−^^2^), as summarized in Figure 3i.

**FIGURE 4:**
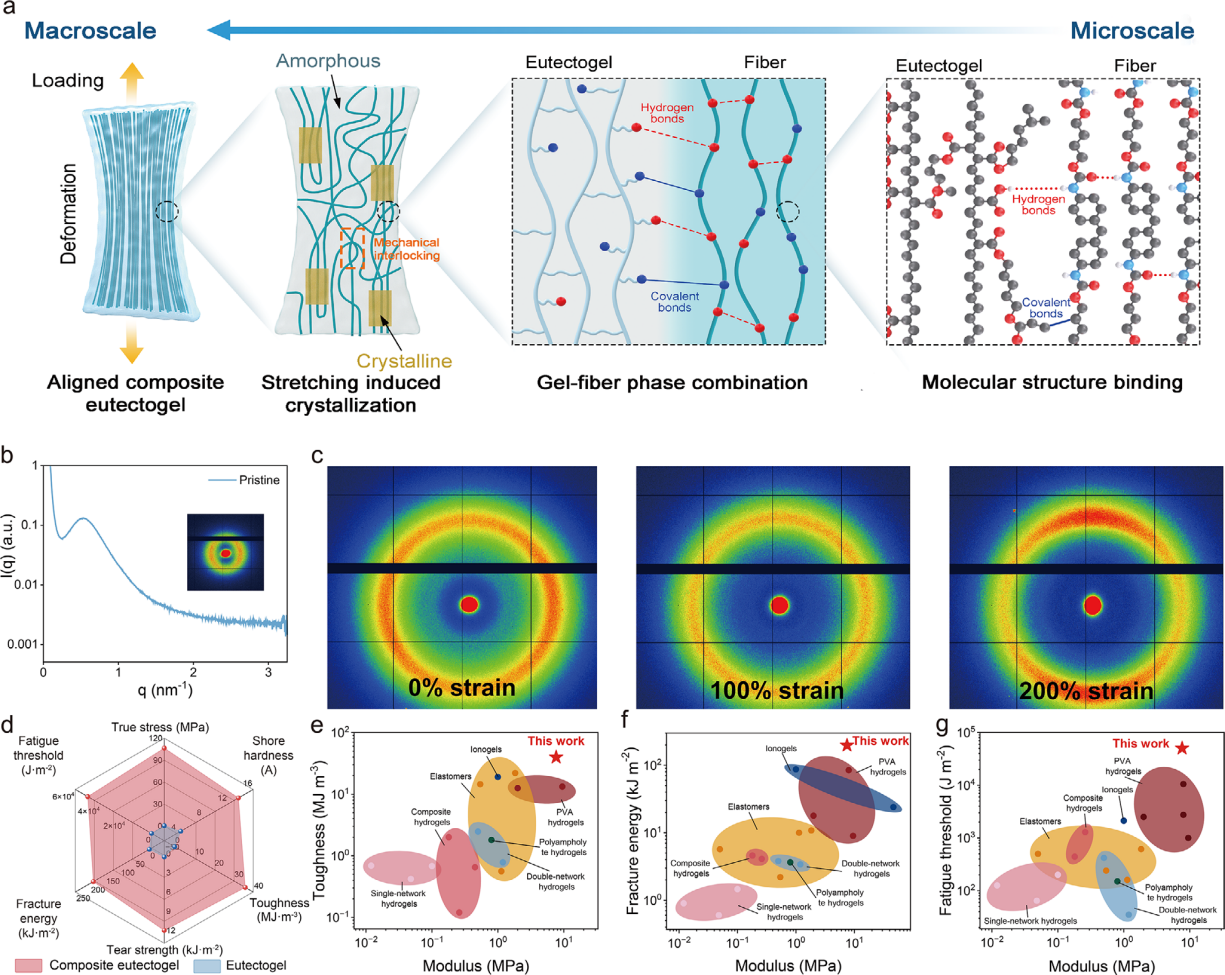
Fiber reinforcement mechanism. (a) Fiber reinforcement and interface bonding mechanism. (b) SAXS curve of composite eutectogel (inset: 2D SAXS pattern). (c) In situ tensile WAXD patterns at different elongations (0%, 100%, and 200%). (d) Comparison of comprehensive mechanical performance between composite and pure eutectogels. Comparison of (e) Toughness‐Modulus, (f) fracture energy‐Modulus and (g) fatigue threshold‐Modulus diagrams with recently‐reported elastomers and gel materials.

Figure 4a schematically illustrates the fatigue‐resistant mechanism of the fiber‐reinforced eutectogel. In the relaxed state, strong interfacial adhesion between the eutectogel and PU fibers is provided by a combination of micro–nano mechanical interlocking, interfacial covalent bonding, and hydrogen bonding between the carboxyl groups ─COOH) in the eutectogel and the urethane groups (─NH─CO─O─) of the PU fibers (Figures S7 and S8). Upon stretching near a notch, fiber alignment promotes a more uniform and continuous fiber–gel interface along the loading direction, facilitating efficient load transfer and progressive hydrogen‐bond dissociation and reformation. This cooperative interfacial interaction dissipates mechanical energy and suppresses localized debonding, thereby enhancing resistance to crack propagation under cyclic loading.

Simultaneously, stretching‐induced crystallization is promoted, leading to an increased crystallinity of the fiber reinforcement. Small‐angle X‐ray scattering (SAXS) and Wide‐angle X‐ray diffraction (WAXD) were further employed to investigate the structural evolution underlying this reinforcement (Figure 4b,c). The SAXS profile exhibits a sharp scattering peak at 0.53 nm^−^^1^, which corresponds to a characteristic structural periodicity of 11.8 nm according to the Bragg equation (d = 2π/q), indicating the presence of a periodic microphase‐separated nanostructure associated with the PU reinforcement. Mechanical resilience typically requires such a hard–soft domain architecture. In addition, WAXD patterns indicate that the crystalline diffraction signals of the composite become more pronounced with increasing elongation. The same trend is observed in the pure PU skeleton, demonstrating that stretching induces molecular alignment and crystallization (Figures S9 and S10). Strain‐induced crystallization therefore provides an effective mechanism for energy dissipation, which helps inhibit catastrophic failure.

Direct comparison between the pure eutectogel and the composite eutectogel confirms that the composite exhibits substantially improved mechanical performance and fatigue resistance (Figure 4d). To place this performance in context, we compared the material with recently reported gel materials and elastomers by plotting fracture toughness, fracture energy, and fatigue threshold as a function of Young's modulus (Figure 4e,g and Table S3). The comparison shows that both fracture energy and fatigue threshold of the composite eutectogel are enhanced by more than an order of magnitude. These values are significantly higher than those reported for state‐of‐the‐art antifatigue hydrogels, ionogels, and elastomers. This exceptional resistance to crack propagation and cyclic damage highlights the potential of the eutectic skin as a reliable platform for mechanically demanding wearable applications.

### 2.4 Adhesion Performance

**FIGURE 5:**
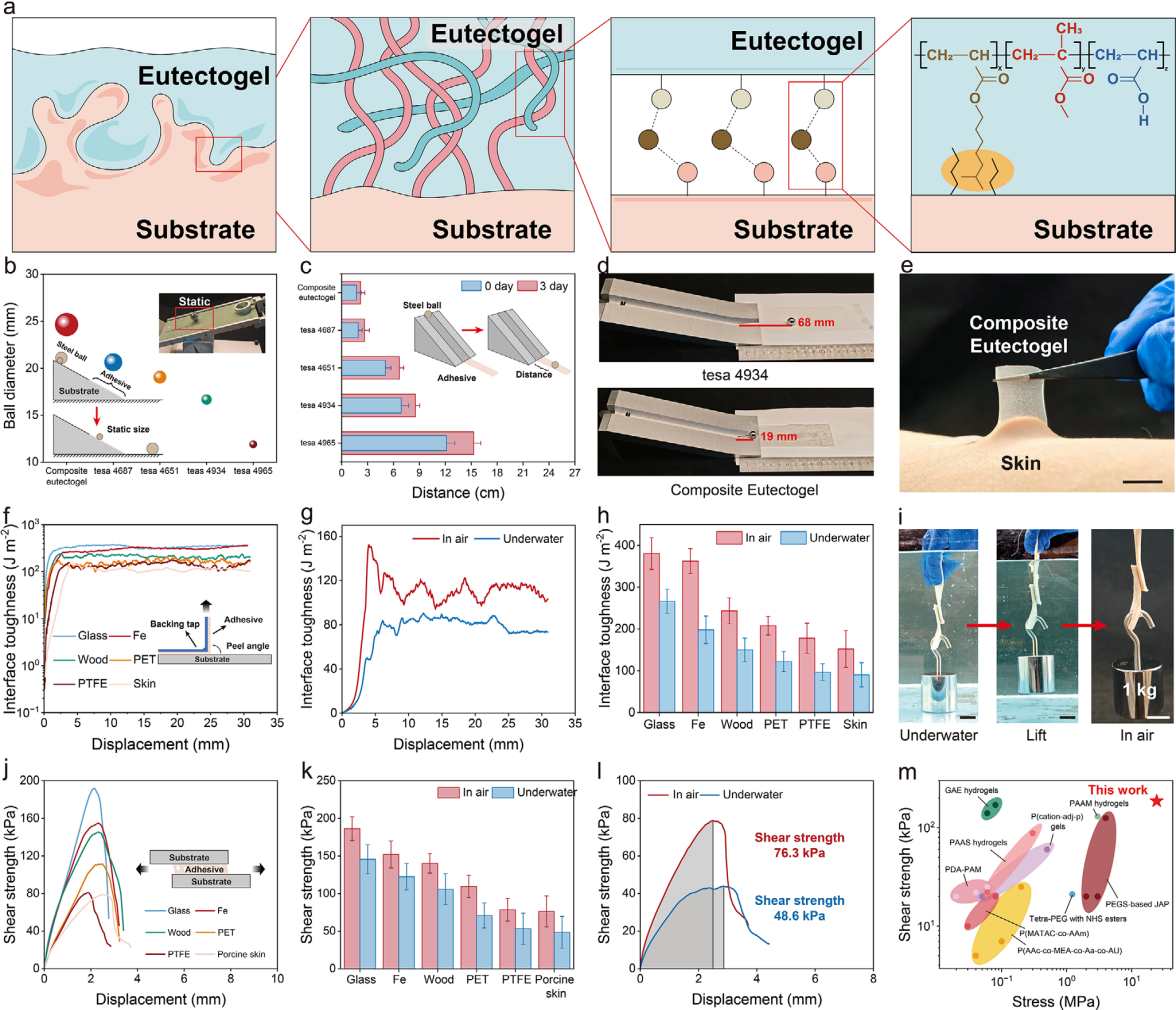
Adhesion performance of composite eutectogel. (a) Schematic diagram of the adhesion mechanism of composite eutectogel. Rolling ball test with (b) different ball sizes and (c) varying distances. (d) Comparison of composite eutectogel and commercial adhesive. (e) Demonstration of skin adhesion using composite eutectogel (Scale bar: 1 cm). (f) Interfacial toughness of composite eutectogels on different substrates. (g) 90° peel curves conducted in air and underwater. (h) Comparison of interfacial toughness in air and underwater among different substrates. (i) Lifting a 1 kg weight in different environments. (Scale bar: 1 cm). Shear adhesion curves (j) and peak values comparison (k) on diverse substrates. (l) Shear curves conducted in air and underwater. (m) Comparison of shear adhesion with recently reported gels.

Figure 5a demonstrates that eutectogels achieve strong adhesion by forming robust mechanical interlocking with the adherend surface. Poly (isooctyl acrylate), with its long alkyl side chains, can permeate microscopic surface depressions of the substrate under gentle pressure, creating an effective anchoring effect. Simultaneously, the acrylic moieties and HES components enhance hydrogen bonding with the adherend. The MMA segments further increase the cohesive energy of the bulk material, thereby suppressing cohesive failure during peeling [60, 61]. In addition, the intrinsic hydrophobicity of the eutectogel prevents moisture‐induced weakening, enabling intimate interfacial contact and durable adhesion even in moist environments.

Initial tack is particularly critical for skin‐mounted wearable devices, as it determines whether an adhesive can achieve rapid and firm attachment to dynamic, irregular skin surfaces without prolonged pressure [62]. To evaluate this property, the tackiness of the composite eutectogel was experimentally assessed and compared with commercially available adhesives using ball‐rolling tests (Note S1). As shown in Figure 5b,c and Videos S1–S4, the composite eutectogel exhibits a strong immediate braking effect, stopping a heavy rolling steel ball (diameter: 24.6 mm) within a very short distance of 1.7 mm. In contrast, commercial adhesives require substantially longer stopping distances of 1.9–12 mm to arrest considerably lighter balls (diameter: 11–20 mm). A direct visual comparison with Tesa 4934 adhesive tape further highlights this performance advantage (Figure 5d).

The reduced stopping distance reflects faster bonding kinetics and rapid intermolecular interactions upon contact. This high instantaneous adhesion enables the composite eutectogel to effectively arrest heavy objects over short distances. The low glass transition temperature (Tg = −45.6°C; Figure S11) facilitates polymer chain mobility, allowing rapid surface wetting and the formation of microscopic mechanical interlocks [63]. Such molecular responsiveness underlies the excellent tackiness of the material, making it highly promising for skin interfaces that require immediate, comfortable adhesion.

To systematically evaluate the dependence of adhesion on substrate surface energy, 90° peel tests were performed on a range of substrates with different surface energies (Table S4). As shown in Figure 5e, the composite eutectogel maintains stable contact with human skin under deformation. Figure 5f further demonstrates that the eutectogel exhibits strong adhesion to diverse substrates, including glass, iron, wood, polyethylene terephthalate (PET), and polytetrafluoroethylene (PTFE). Notably, the adhesion energy on skin reaches 152.2 J m^−^^2^, indicating reliable and durable bonding.

Adhesion in aqueous environments is generally challenging because interfacial water films hinder intermolecular interactions [64]. In contrast, the intrinsically hydrophobic eutectogel limits water penetration at the interface, thereby preserving adhesive bonding. As shown in Figure 5g,h, the eutectogel exhibits high interfacial toughness on various substrates in both air and underwater environments. For example, the adhesion energy on glass reaches 380.6 J m^−^^2^ in air and remains 266 J m^−^^2^ in water. Strong adhesion to porcine skin under aqueous conditions is further demonstrated in Figure 5i and Video S5. Consistently, lap shear tests (Figure 5j–l) show that the eutectogel retains a shear strength of 48.6 kPa on wet porcine skin. Suppression of interfacial wetting and water diffusion by hydrophobic segments prevents debonding, while hydrophobic interactions and molecular chain entanglement enable stable stress transfer and resistance to shear forces during submergence. Meanwhile, the adhesion performance of the composite eutectogel exhibits no noticeable decline at different temperatures (25°C, 37°C, and 50°C). This proves that high temperatures exert a negligible influence on its interfacial adhesion properties and internal cohesive energy (Figures S12 and S13).

We compared the composite eutectogel with reported health‐monitoring gel materials, as summarized in Figure 5m and Table S5. This comparison indicates that the composite eutectogel exhibits clear advantages over existing gel materials in terms of mechanical robustness and wet adhesion. This performance arises from the synergistic contributions of the HES and acrylate segments. The stability of the eutectogel across diverse environments highlights its significant potential as a next‐generation soft material for wearable electronic applications operating under harsh conditions.

### 2.5 High‐Fidelity and Long‐Term Electrophysiological Monitoring

**FIGURE 6:**
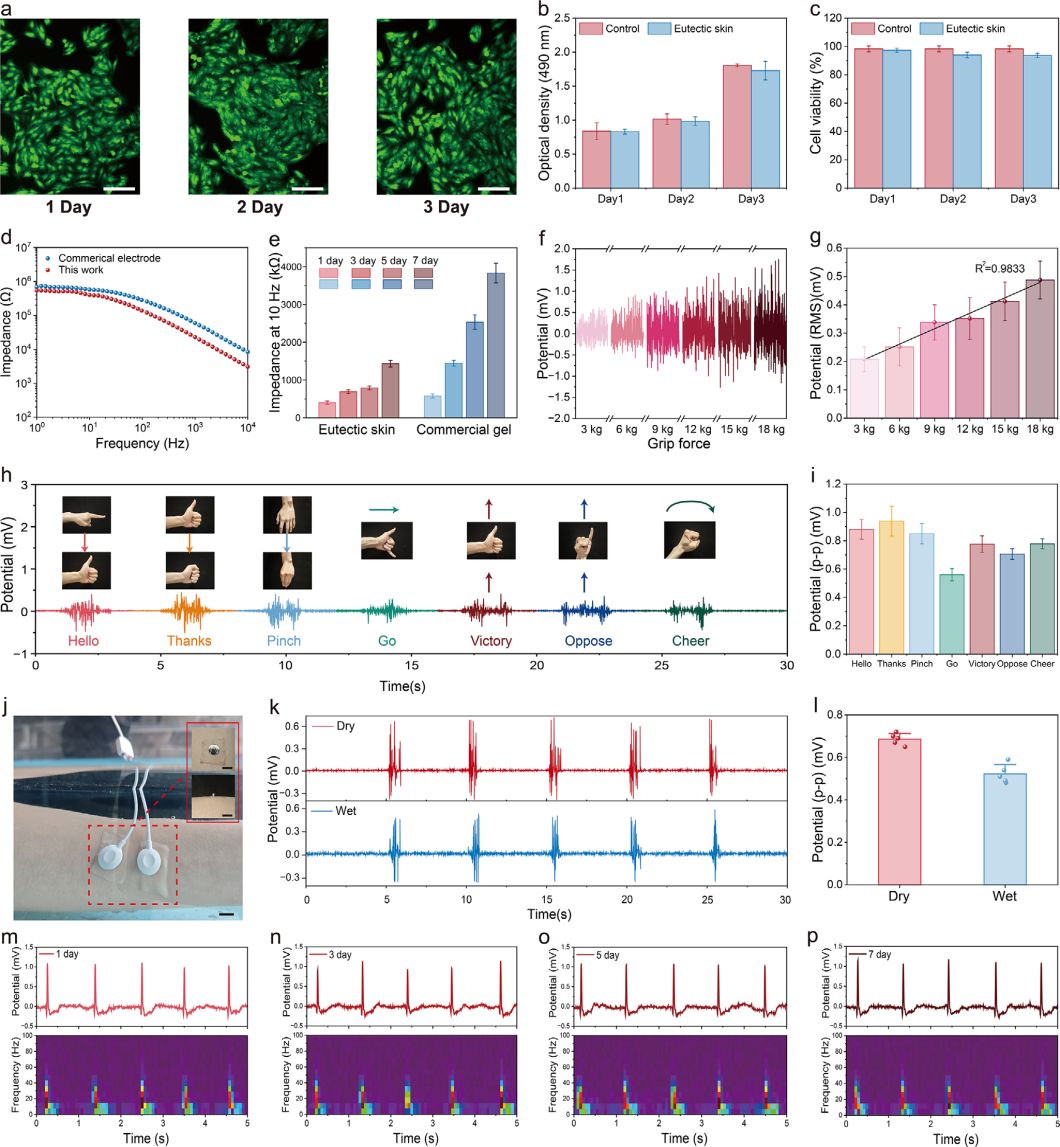
Application of eutectic skin in health monitoring. (a) Fluorescence cell images of composite eutectogel at 1–3 day (Scale bar: 100 µm). (b) Optical density and (c) cell viability of composite eutectogel and control group at different days. (d,e) Interfacial impedance at different times. (f) Detection of EMG signals under different grip. (g) Potential and RMS value under different grip force. (h) EMG signals and (i) potential for different sign languages. (j) Underwater EMG demonstration (Scale bar: 1 cm). (k) EMG signals and (l) potential in air and underwater. (m–p) ECG signals and time‐frequency diagrams at different days (1–7 day).

We refer to the strong, skin‐analogous ionic interface formed by the composite eutectogel as “eutectic skin.” This material combines excellent biocompatibility with strong adhesion and fatigue resistance. Live/dead fluorescence images (Figure 6a and Figure S14) show well‐spread cells with normal morphology and minimal cell death, comparable to the negative control. Cell‐counting‐kit‐8 (CCK‐8) assays further confirm negligible cytotoxicity, as shown in Figure 6b,c, with cell viabilities of approximately 97% and 93% after 24 and 72 h, respectively. This biological safety complements the material's intrinsic ionic conductivity and mechanical stability. As a result, the eutectic skin provides a stable platform for signal acquisition, outperforming conventional ionic skins that are often limited by swelling or loss of adhesion.

Figure 6d,e presents electrochemical impedance spectroscopy data for the eutectic skin and commercial gel electrodes. At 10 Hz, the eutectic skin exhibits an impedance of 399.4 kΩ (Figure 6d), which is substantially lower than the 576.6 kΩ measured for commercial Ag/AgCl electrodes. Durability was further evaluated over a 7‐day period. During this time, the impedance of the eutectic skin increased moderately from 399.4 to 1432.8 kΩ, whereas the commercial gel electrode showed pronounced degradation, with impedance rising from 576.6 to 3831.5 kΩ (Figure 6e and Figure S15). These results confirm that the eutectic skin maintains a stable conductive network and interfacial integrity during prolonged use, providing a solid foundation for high‐fidelity physiological monitoring.

Owing to its biocompatibility, high ionic conductivity, strong adhesion, and fatigue resistance, the eutectic skin enables high‐fidelity electromyography (EMG) monitoring in humans. These properties allow reliable extraction of low‐amplitude signals with high accuracy, enabling clear discrimination of individual sign‐language gestures. Sequential grip tests ranging from 3 to 18 kg show consistent responses to dynamic muscle activity, with root‐mean‐square EMG analysis confirming stable signal quality across varying force levels (Figure 6f,g).

Under complex physiological motions, the eutectic skin provides enhanced signal resolution and robust capture of gesture‐specific EMG patterns (Figure 6h,i and Figure S16). Notably, its hydrophobicity and interfacial stability enable reliable operation under aqueous conditions. At a water depth of 20 cm and 25°C, the eutectic skin maintains firm skin adhesion, suppresses water interference, and records clear EMG signals during fist clenching (Figure 6j–l and Video S6), achieving a signal‐to‐noise ratio (SNR) of ∼20 dB (Figure S17), comparable to that of SNR value (27.9 dB) measured in the dry environment (Table S6). These results highlight the eutectic skin as a durable and versatile platform for high‐accuracy electrophysiological monitoring in both dry and wet environments.

In addition, continuous electrocardiogram (ECG) monitoring was performed to evaluate long‐term signal stability. The recorded ECG waveforms remain stable and consistent as the monitoring duration increases. Notably, over a 7‐day continuous test, no signal discontinuity or abnormal waveform distortion was observed across the relevant frequency range (0.05–100 Hz) (Figure 6m–p and Figure S18). These results demonstrate that the eutectogel provides reliable and consistent ECG measurements during extended wear, fulfilling key requirements for long‐term health monitoring.

Collectively, the eutectic skin integrates wet stability, ionic conductivity, and fatigue resistance, offering a distinct advantage over conventional ionic skins and underscoring its potential as a next‐generation interface for diverse electrophysiological applications.

## 3 Conclusions

In summary, we have developed a fatigue‐resistant eutectic skin inspired by the hierarchical support architecture of *Acorus calamus* leaves. The long‐standing trade‐off between high mechanical strength and tissue‐like softness is resolved through a rational fiber‐reinforcement strategy. An aligned polyurethane skeleton enables efficient energy dissipation and suppression of crack propagation, imparting exceptional fatigue resistance and mechanical longevity. Intrinsic hydrophobicity of the eutectogel matrix further ensures strong wet adhesion and environmental stability on biological tissues. The demonstrated high‐fidelity signal acquisition under prolonged wear and in aquatic environments confirms the practical applicability of this system. We anticipate that this work establishes a generalizable design paradigm for mechanically resilient and environmentally stable soft interfaces, paving the way for next‐generation wearable bioelectronics capable of reliable operation under dynamic and extreme conditions.

## 4 Experimental Section

### 4.1 Materials

All chemicals were used without any further purification. Isooctyl acrylate (IA, 99%), poly(ethylene glycol) diacrylate (PEGDA, average Mn 700), acrylic acid (AA), methyl methacrylate (MMA), L‐(‐)‐Menthol (Men), N‐Octanoic acid (OA), Benzophenone, 2‐(Acryloyloxy)‐N,N,N‐Trimethylethanaminium chloride (80 wt.% in H_2_O), 1‐ethyl‐3‐methylimidazolium bis (Trifluoromethylsulfonyl) imide, and 2‐hydroxy‐4′‐(2‐hydroxyethoxy)‐2‐methylpropiophenone (PI 2959) were purchased from Adamas. Tetrahydrofuran (THF), N,N′‐Dimethylformamide (DMF) were purchased from Sinopharm Chemical Reagent Co., Ltd. Polyurethane (PU) was purchased from BASF.

### 4.2 Hydrophobic Eutectic Solvents Preparation

Hydrophobic eutectic solvents (HES) were prepared by the heating method, based on mixing the three components (menthol, chloride, and organic acids) and heating them at 65°C under constant stirring until a homogeneous liquid is formed.

### 4.3 Aligned Fiber Skeleton Preparation and Surface Functionalization

Aligned fiber skeleton was prepared by electrospinning. First, PU is dissolved in a mixed solvent of DMF and THF (1/1, V/V) to prepare an electrospinning solution with a solid content of 22 wt.% at 80°C, with 1‐ethyl‐3‐methylimidazolium bis (trifluoromethylsulfonyl) imide to enhance conductivity. The control voltage was set to 15 kV, using a 25G needle with an injection rate of 0.01 mL·min^−1^. The receiving roller speed was adjusted to 1000 rpm to obtain an aligned fiber skeleton. Thereafter, the fiber skeleton was subjected to heat treatment in an oven at 105°C, followed by surface modification in a methanol solution containing 0.1 wt.% benzophenone. After drying, an aligned surface‐functionalized fiber skeleton was obtained.

### 4.4 Pure Eutectogels Synthesis

Eutectogel precursor is prepared by combining HES with monomers. The HES were mixed with IA, AA, MMA, PEGDA (1 wt.% based on monomers), and the PI 2959 (0.1 wt.% based on monomers). The precursor was vortexed for a few seconds, poured into PTFE molds, and irradiated with a 400 mW·cm^−2^ lamp intensity for 60 min to obtain the pure eutectogel.

### 4.5 Composite Eutectogels Synthesis

Surface‐functionalized aligned fiber skeleton was immersed in the eutectogel precursor, and irradiated with a 400 mW·cm^−2^ lamp intensity. Then, the eutectogel was soaked in HES for 3 days to remove unreacted monomers.

### 4.6 General Characterizations

The microstructure of the eutectogel was characterized by field emission scanning electron microscopy (Hitachi, SU8600) and optical microscopy (Evident BX53F2C). The surface roughness of the composite eutectogel and the pure eutectogel was analyzed by using atomic force microscopy (Bruker Dimension ICON). The transmittance of eutectogel was analyzed by using UV–vis spectrophotometer (TU1950). The contact angle test was carried out by an optical contact goniometer (Biolin, Theta Flow). The volume of the water droplets during each measurement was maintained at 20 µL, and at least five measuring points were taken on each sample surface to obtain the final average contact angle. DSC tests were conducted on a Mettler Toledo DSC instrument within a temperature range of −80°C to 30°C in a nitrogen atmosphere at a heating rate of 10°C/min. Heat the test process twice in a cycle to eliminate the thermal history and the influence of other factors. The initial mass of the gel was recorded as M g after drying. Put the sample into water. After the preset time, take out the sample and gently absorb the excess water on the surface. The mass of the swollen sample was weighed using a high‐precision balance and recorded as M1 g. Calculate the weight swelling rate (%) through the formula:

$$Weight\;swelling\;ratio\;\left(\%\right)=\left(M_{1}-M\right)/M\times 100\%$$

SAXS and WAXD measurements of the eutectogel were conducted on Xeuss 3.0HR, with X‐ray energy of 10.0 keV and wavelength of 1.24 A. During measurement, place the sample perpendicular to the beam. The distance from the sample to the detector is 1.87 m, covering the scattering vector q (q = (4π/λ)sin(θ) of 0.1–5 nm^−1^; 2θ is the scattering Angle. The acquisition time of the light scattering pattern is 180 s. The intensity distribution is obtained by the radial average of the SAXS diagram in the horizontal direction. To meet the acquisition requirements of wide‐angle scattered signals, the distance from the sample to the detector was adjusted to 0.20m. Combined with the optical configuration of the instrument, the scattering vector q range of 5.0–30.0 nm^−^^1^ was ultimately covered, and the intensity distribution of the WAXD map in the horizontal direction was obtained.

### 4.7 Mechanical Characterization

The mechanical properties of eutectogel were characterized using the MTS e42.503 universal testing machine equipped with a 250 N sensor at room temperature (relative humidity 60 ± 1%, 25°C). Strength assessment is conducted using real stress (calculated by multiplying the nominal stress by the deformation rate based on the incompressibility assumption). Toughness is calculated from the integral area of the nominal stress‐strain curve to the fracture point. To conduct the tear test, the eutectogel was cut into trouser samples and tested at a tensile rate of 50 mm/min. The tearing energy (Γ) can be calculated according to the following formula. F represents the average force at the time of tearing, and d is the thickness of the trouser sample:

Γ=2F/d

The breaking energy (Gc) was measured using Single‐edge notch tension tests (SENT). For samples with notches, use a sharp razor to make a notch in the middle of the test side. The calculation formula for fracture energy is as follows, where λ_c_ is the fracture deformation ratio of the notched specimen (λ_c_ = ε_c_ + 1), c represents the notch length, and W is the strain energy obtained by integrating the stress–strain curve of a specimen of the same size without notches stretched to ε_c_ strain:

$$Gc=\frac{6Wc}{\sqrt{\lambda_{c}}}$$

The fatigue threshold (resistance to fatigue fracture) of the eutectogel was measured by the single‐notch method. Specimens with pre‐cut notches (approximately 1/5 of the width) are subjected to cyclic tensile testing at different strains. The energy release rate (G) of the notched specimen in the NTH cycle is calculated as follows, where k(N) is the strain change function determined by $k\;\left(N\right)=3/\sqrt{\varepsilon +1}$,c(N) is the crack propagation length, and W(N) is the strain energy density of the notched specimen of the same size stretched to the same strain ε. The strain energy density W(N) of the notched specimen in the NTH cycle can be obtained by using $W_{\left(N\right)}=\int_{0}^{\varepsilon }\sigma d\varepsilon$:

$$G\left(N\right)=2k\left(N\right)\cdot c\left(N\right)\cdot W\left(N\right)$$

### 4.8 Adhesion Properties

To characterize the adhesion properties of the eutectogels, lap shear and 90° peeling tests were conducted by an MTS e42.503 universal testing machine equipped with a 250 N load cell. The grease of the porcine skin was removed by a scalpel and the dermal surface of the porcine skin was cleaned by PBS buffer and deionized water sequentially. Other adhered substrates should be directly cleaned with deionized water. For the 90° peel test, the tensile speed is set at 50 mm min‐1. The gel (20 × 150 mm^2^) was adhered to the surface of the substrate and peeled off at a constant speed. The interfacial toughness was determined as the platform force divided by twice the width of the adhered area. Eutectogels (15 × 15 mm^2^) were sandwiched between two pieces of substrate to form the adhesion joint with a gentle pressure of 5 kPa for 20 s, and the adhesion strength was determined as the maximum force during the shearing process divided by the adhesion area. All the experiments were carried out in the environment chamber with a temperature of 25°C and humidity of 60 ± 1%, and at least 3 samples were tested for each group.

### 4.9 Biocompatibility Characterization

The cytotoxicity of the eutectogel was evaluated by the determination of Cell‐Counting‐Kit‐8 (CCK‐8). HUVECs cells were cultured in a 5% CO_2_ environment at 37°C for 24 h. Subsequently, the original culture medium was replaced with eutectogel for incubation. Add 100 µL of CCK‐8 solution to each well and incubate at 37°C for 1 h. Finally, the absorbance was measured at 450 nm.

### 4.10 Electrophysiological Characterization

The eutectic skin is cut into circular pieces for assembling the skin‐attached electrodes. Biopotential signals (ECG and EMG) were collected by homemade recorders based on the Arduino platform. The signal‐to‐noise ratio (SNR) of the gel is calculated by the following formula, where N is the number of samples and V_signal(k)_ and V_noise(k)_ are the potential values of the signal and noise:

$$SNR\left(dB\right)=20\times log_{10}\frac{\sqrt{\sum_{k=1}^{N}V_{signal\left(k\right)}^{2}}}{\sqrt{\sum_{k=1}^{N}V_{noise\left(k\right)}^{2}}}$$

All experiments involving human participants were conducted in accordance with relevant guidelines and regulations and were strictly approved by the Ethics Committee of the First Affiliated Hospital of Zhengzhou University (Approval No. 2026‐KY‐0667). Informed consent was obtained from all volunteers prior to all human experiments reported in the manuscript.

## Conflicts of Interest

The authors declare no conflicts of interest.

## Supporting information




**Supporting File1**: adma73563‐sup‐0001‐SuppMat.docx.

**Supporting File2**: adma73563‐sup‐0002‐VideoS1.mp4.

**Supporting File3**: adma73563‐sup‐0003‐VideoS2.mp4.

**Supporting File4**: adma73563‐sup‐0004‐VideoS3.mp4.

**Supporting File5**: adma73563‐sup‐0005‐VideoS4.mp4.

**Supporting File6**: adma73563‐sup‐0006‐VideoS5.mp4.

**Supporting File7**: adma73563‐sup‐0007‐VideoS6.mp4.

## Data Availability

The data that support the findings of this study are available from the corresponding author upon reasonable request.
