# Supplementary material for: Leaf‐Inspired Eutectic Skin With Extreme Fatigue Resistance and Robust Wet Adhesion for Amphibious Epidermal Electronics
Source: Adv Mater. 2026 May 29;38(37):e73563. doi: 10.1002/adma.73563 (PMC13327141; doi:10.1002/adma.73563)
Supplement: Supplementary file 1 — Supporting File1: adma73563‐sup‐0001‐SuppMat.docx. [file ADMA-38-e73563-s007.docx]

**Supporting Information**

**Leaf-Inspired Eutectic Skin with Extreme Fatigue Resistance and Robust Wet Adhesion for Amphibious Epidermal Electronics**

Jiayu Hou, Jiancheng Dong*, Je Hyeong Kim, Chang Zhou, Shiyin Lin, Xingyu Liu, Hao Qiu, Mengting Zheng, Yuduo Zhang, Haijun Zhu, Kangjia Geng, Yidong Peng, Haoran Liu, Yunpeng Huang, Yongsheng Luo, Steve Park*, and Tianxi Liu*

Jiayu Hou, Jiancheng Dong, Chang Zhou, Shiyin Lin, Xingyu Liu, Hao Qiu, Mengting Zheng, Yuduo Zhang, Haijun Zhu, Kangjia Geng, Yidong Peng, Haoran Liu, Yunpeng Huang, and Tianxi Liu

Key Laboratory of Synthetic and Biological Colloids, Ministry of Education, School of Chemical and Material Engineering, Jiangnan University, Wuxi 214122, China

Je Hyeong Kim, and Steve Park

Department of Materials Science and Engineering, Korea Advanced Institute of Science and Technology (KAIST), 291 Daehak-ro, Yuseong-gu, Daejeon, 34141, Republic of Korea

Yongsheng Luo

Kidney Transplantation Unit, The First Affiliated Hospital of Zhengzhou University, Zhengzhou 450052, China

**Corresponding Authors**

Jiancheng Dong (email: [jcdong@jiangnan.edu.cn](mailto:jcdong@jiangnan.edu.cn))

Steve Park (email: [stevepark@kaist.ac.kr](mailto:stevepark@kaist.ac.kr))

Tianxi Liu (email: [txliu@jiangnan.edu.cn](mailto:txliu@jiangnan.edu.cn))

**The Supplementary Information file includes:**

Supplementary Note 1

Figure S1 to S18

Table S1 to S6

Legends for Video S1 to S6

**Other supplementary materials for this manuscript include the following:**

Video S1 to S6

**Supplementary Note 1: Ball Rolling Tack Test Procedure**

The initial tackiness of the adhesives was measured according to the ASTM D3121 *Standard Test Method for Ball Tack of Pressure-Sensitive Tapes* to comprehensively evaluate the instantaneous adhesion characteristics of the composite eutectogels.

**Limiting Ball Diameter Stop Test**

***Objective:*** This test assesses the instantaneous adhesive strength by determining the largest steel ball diameter (Note Table S1) that the adhesive can fully arrest when released on an inclined plane. The result directly indicates the material's ability to resist the kinetic energy of a large, heavy sphere upon initial contact (Note Figure S2).


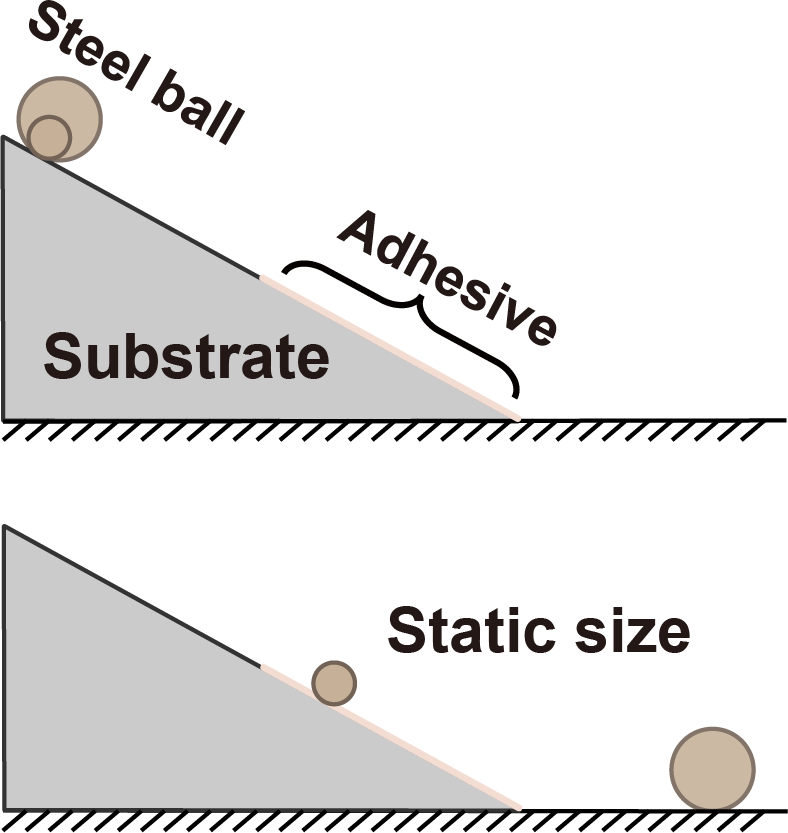


Note Figure S1. Illustration of limiting ball diameter stop test.

***Procedure:***

Test samples were prepared according to specifications and conditioned under the standard environment of 23±2 °C and 50±5% relative humidity. Samples, approximately 25 mm×125 mm in size, were secured to the lower section of the inclined test plane with the adhesive surface facing upward. Strict care was taken to ensure the surface was free of any bubbles, wrinkles, or impurities.

The inclination angle of the test bench was precisely calibrated to the standard 30°. Steel balls of corresponding diameters were selected based on an estimation of the sample's tackiness. The steel ball was gently placed at the starting position at the top of the test bench, ensuring the center was aligned with the track axis.

The steel ball was then released with zero initial velocity and allowed to roll freely down the incline. The linear distance from the stopping point of the ball to the starting edge of the sample was recorded. This procedure was repeated with progressively larger balls until a diameter was found that the sample could no longer stop. A maximum of three trials were conducted for each sample, and the largest diameter successfully stopped was noted. The results informed the discussion regarding the superior "immediate blocking effects" of the eutectic skin mentioned in Section 2.4.

**Kinetic Energy Decay Distance Test**

***Objective:*** This test evaluates the adhesive's contact and bonding kinetics by measuring the distance a pre-accelerated steel ball travels before stopping on the horizontal adhesive surface. A shorter distance signifies faster molecular wetting and greater energy dissipation capability (Note Figure S2).


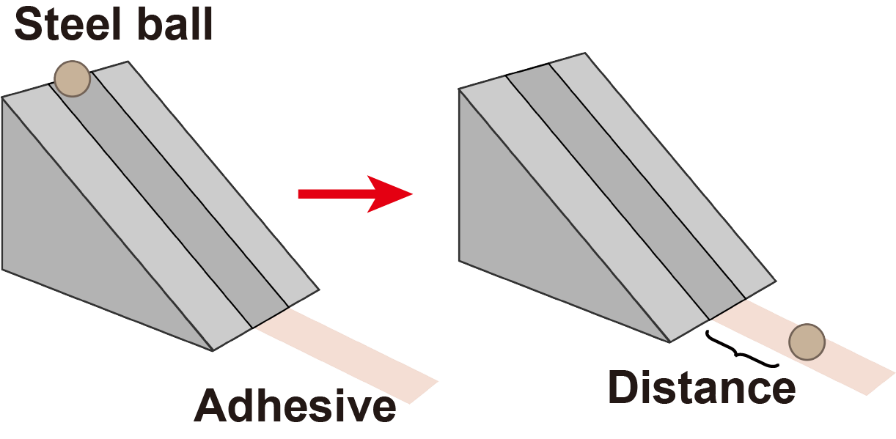


Note Figure S2. Illustration of kinetic energy decay distance test

***Procedure:***

Test specimens, also measuring approximately 25 mm×125 mm, were prepared under standard environmental conditions. The specimen was securely fixed to the horizontal surface immediately following a 21.5° inclined groove. This groove serves to impart a consistent, stable initial kinetic energy to the rolling sphere.

A specific steel ball with a diameter of 7/16 inch (11.113 mm, Note Table S1) was utilized for this test. The ball was gently placed at the starting position and released with zero initial velocity. Upon exiting the 21.5° inclined groove, the ball possessed a fixed velocity as it entered the horizontal adhesive region. The linear distance from the end of the inclined groove (the start of the adhesive contact) to the ball's final stopping point was recorded.

A shorter stopping distance demonstrates superior tasckiness, indicating that the adhesive establishes rapid intermolecular interactions and quickly dissipates the ball's kinetic energy. This result supports the claim in Section 2.4 that the eutectic skin's low glass transition temperature facilitates quick wetting and molecular chain mobility, leading to rapid bond formation. At least three trials were performed per sample, and the average distance was reported as the final result.

**Note Table S1**. Size of steel ball

| Number | Diameter (mm) |
| --- | --- |
| Ball 1 | 11.113 |
| Ball 2 | 11.906 |
| Ball 3 | 12.7 |
| Ball 4 | 13.494 |
| Ball 5 | 14.288 |
| Ball 6 | 15.081 |
| Ball 7 | 15.875 |
| Ball 8 | 16.669 |
| Ball 9 | 17.463 |
| Ball 10 | 18.256 |
| Ball 11 | 19.05 |
| Ball 12 | 19.844 |
| Ball 13 | 20.638 |
| Ball 14 | 21.431 |
| Ball 15 | 22.225 |
| Ball 16 | 23.019 |
| Ball 17 | 23.813 |
| Ball 18 | 24.606 |

The density of all the steel balls is 7.8 g·cm^-3^

**Supplementary Figures**


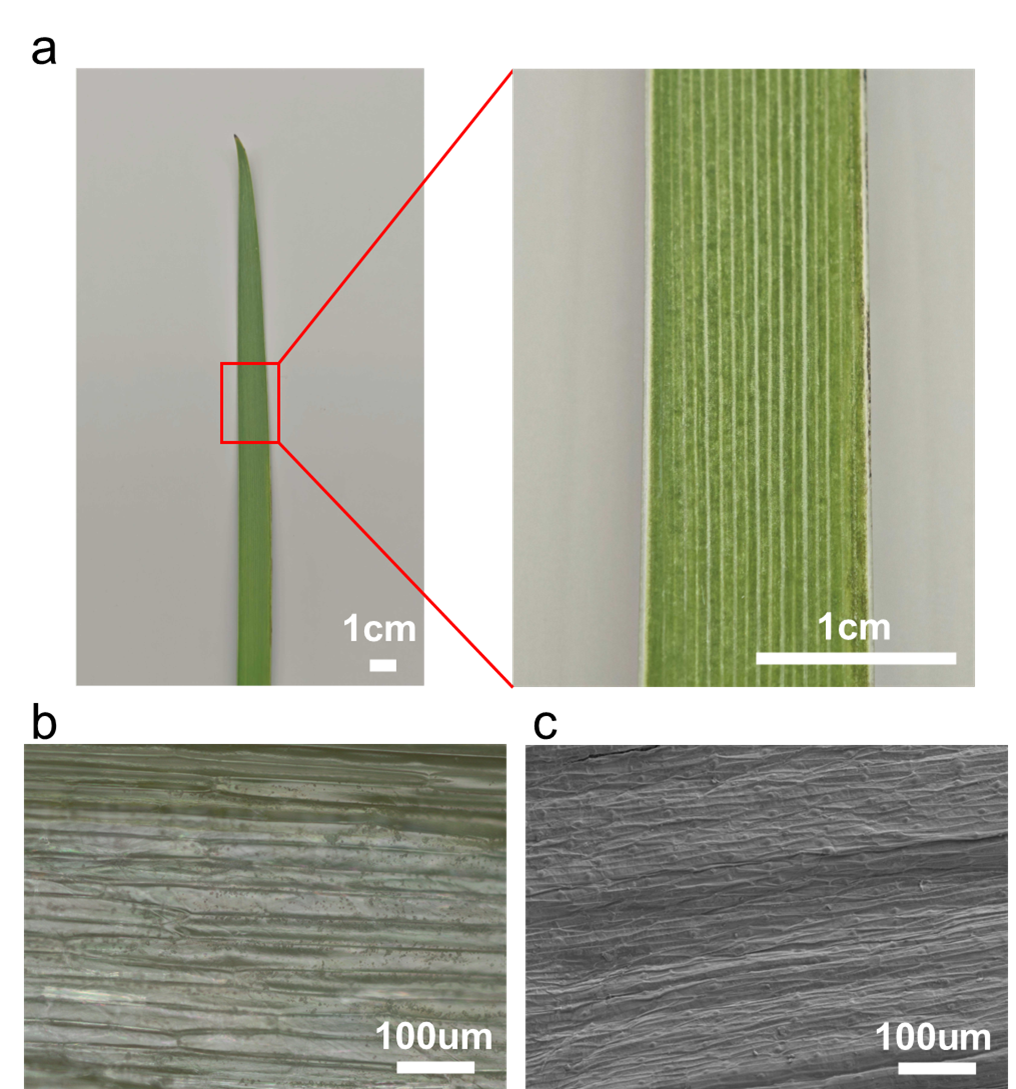


**Figure S1.** (a) Optical images, (b) Optical microscope and (c) SEM image of the leaf structure of *Acorus calamus.*


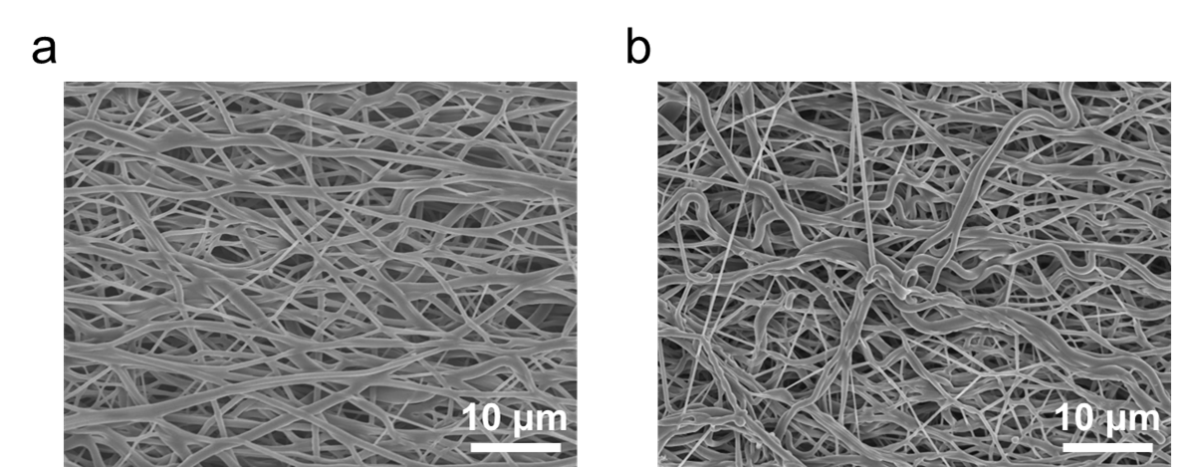


**Figure S2.** SEM images of (a) aligned PU fibers and (b) heat treated PU fibers.


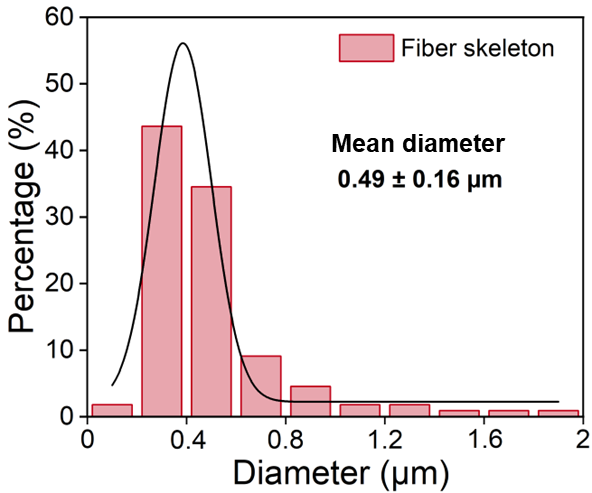


**Figure S3.** The diameter distribution of the aligned fiber skeleton.

**
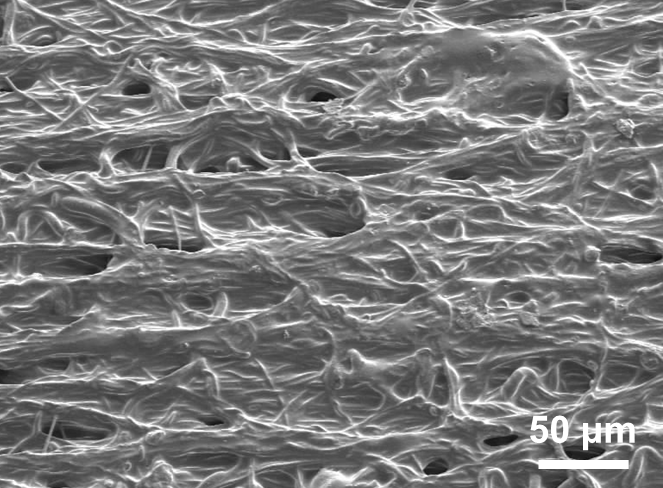
**

**Figure S4.** SEM image of composite eutectogel surface.


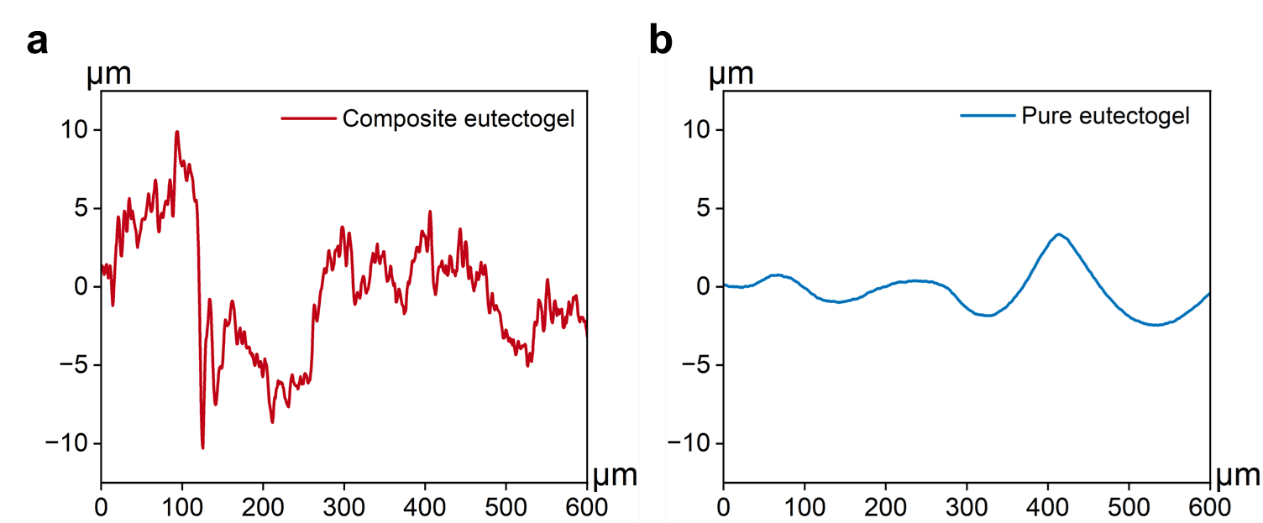


**Figure S5.** Surface roughness of (a) composite eutectogel and (b) pure eutectogel.


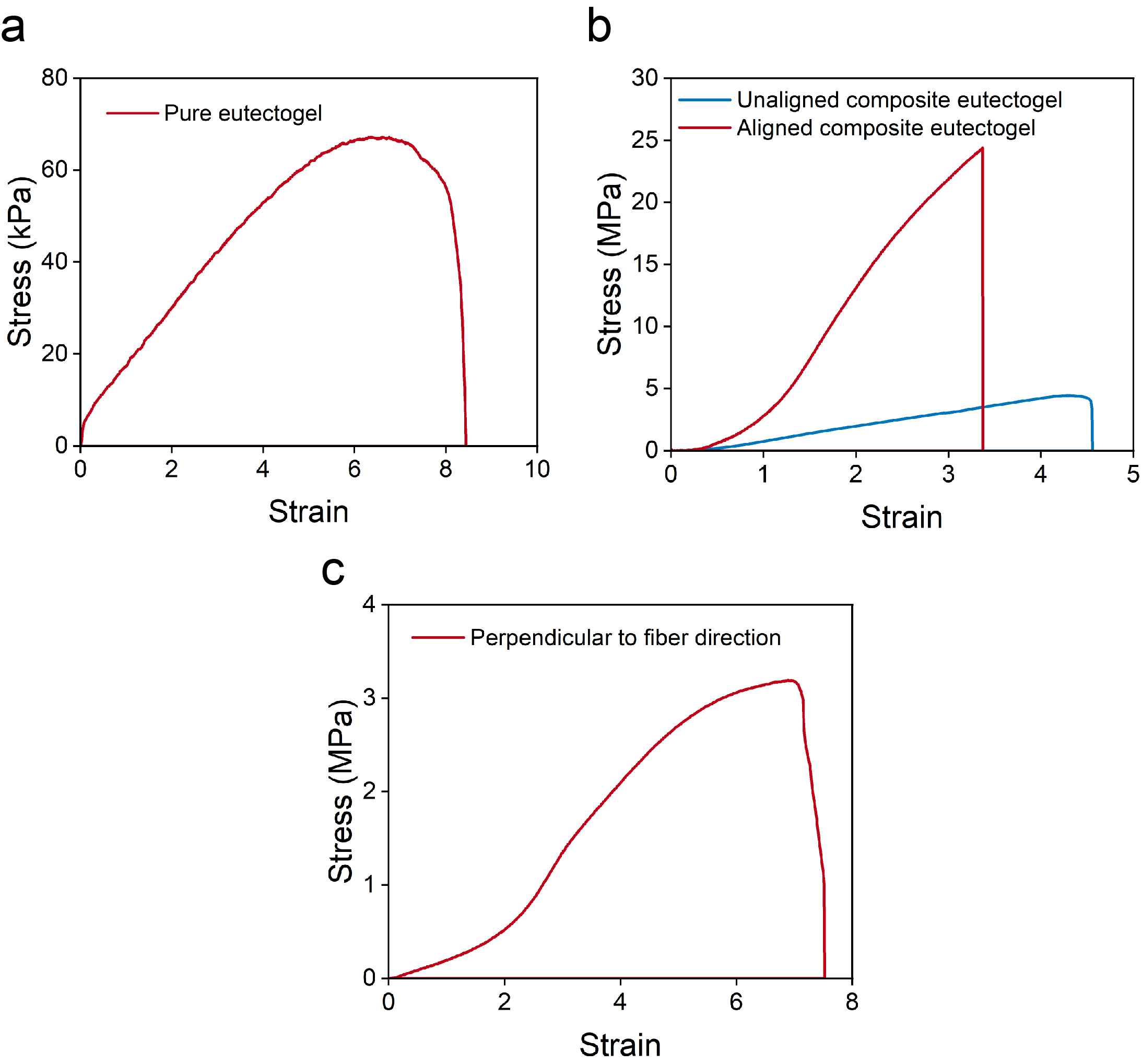


**Figure S6.** Stress-strain curves of (a) pure eutectogel, (b) unaligned and aligned composite eutectogel. (c) Stress-strain curve of composite eutectogel stretched along the perpendicular direction.


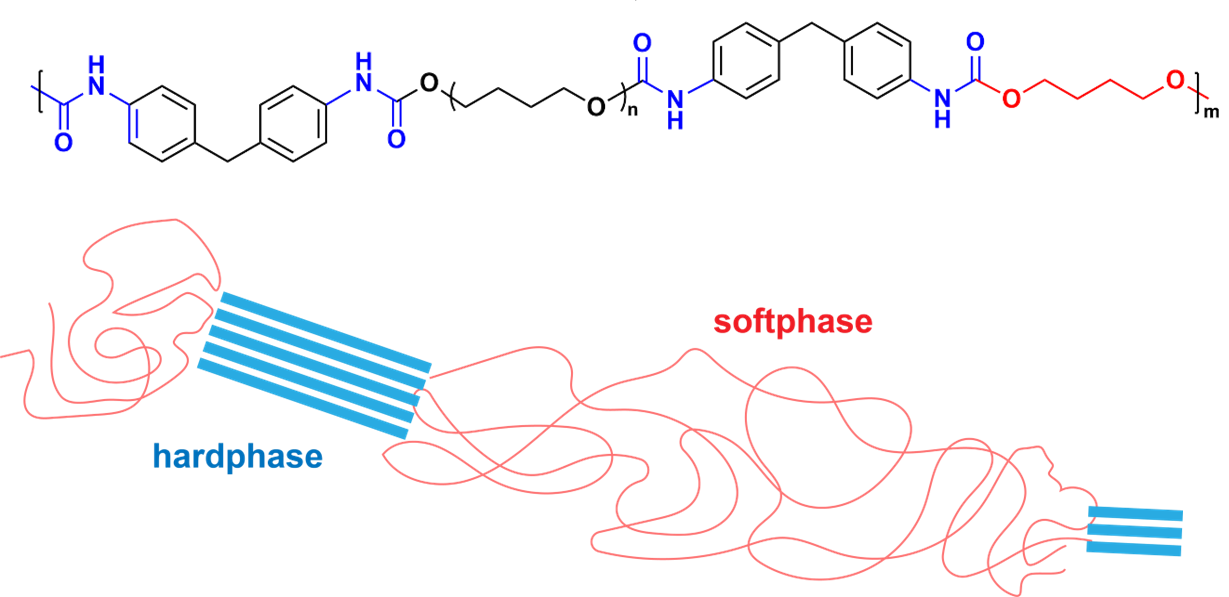


**Figure S7.** Chemical structure of PU elastomer used in this study.


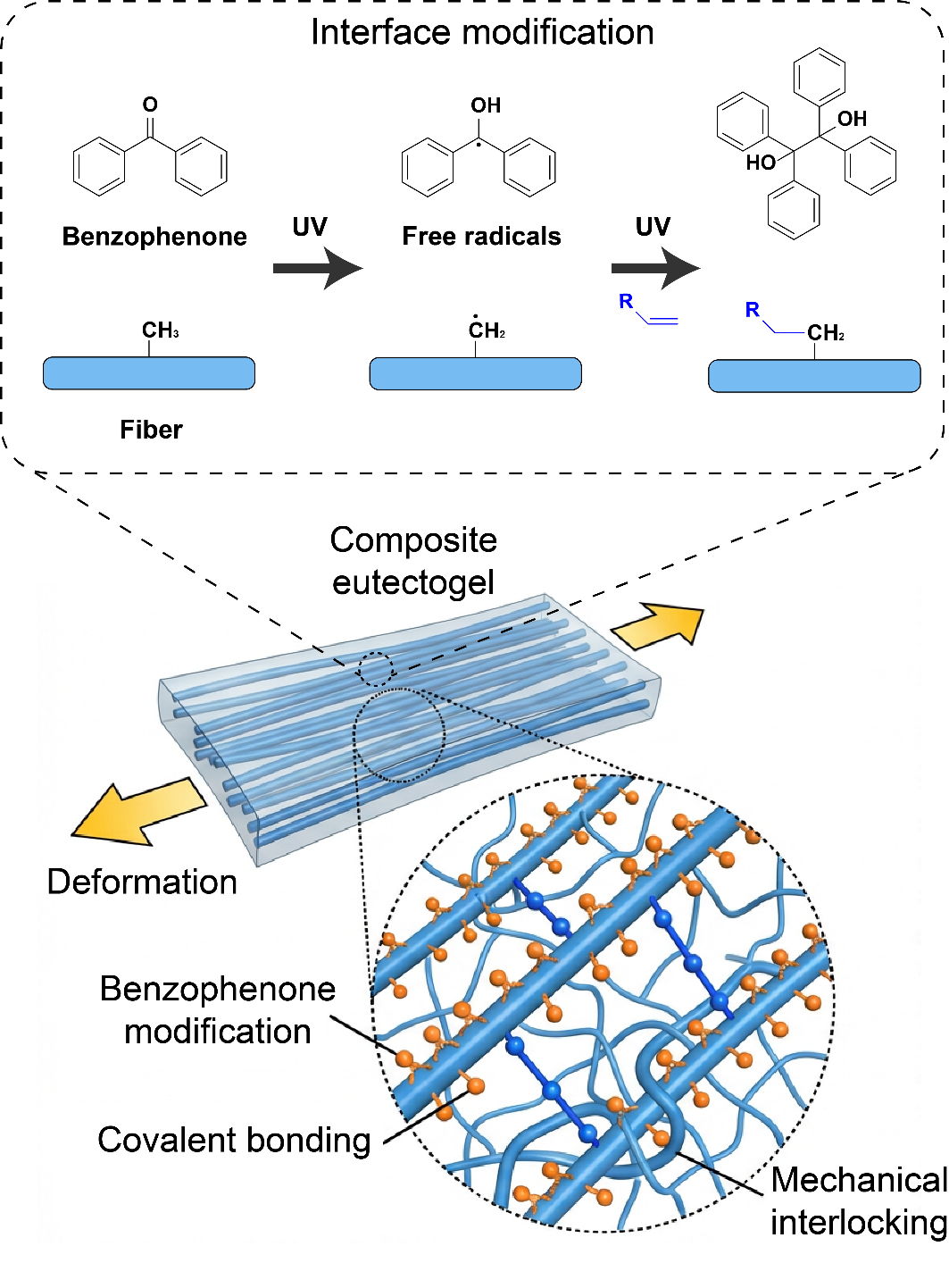


**Figure S8.** Interface modification mechanism of composite eutectogel


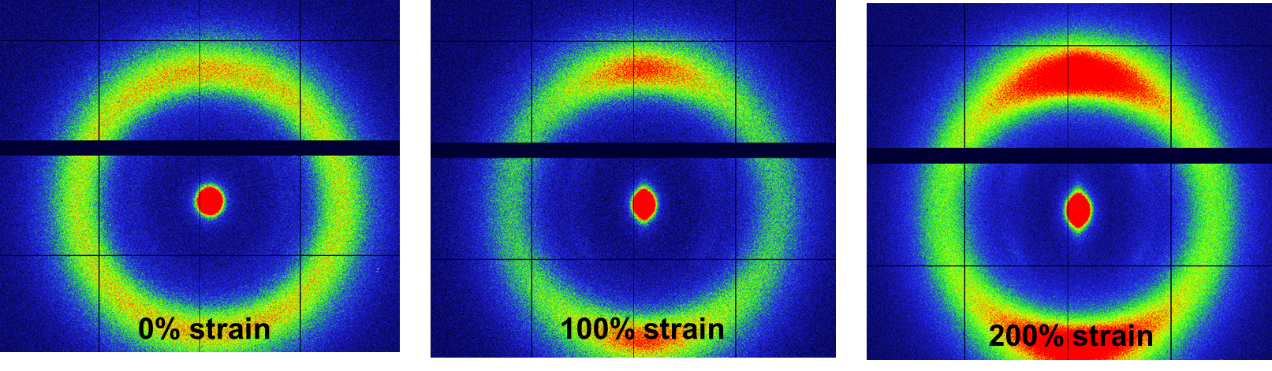


**Figure S9.** WAXD images of in-situ stretching of aligned fiber skeleton (0%, 100%, 200%).


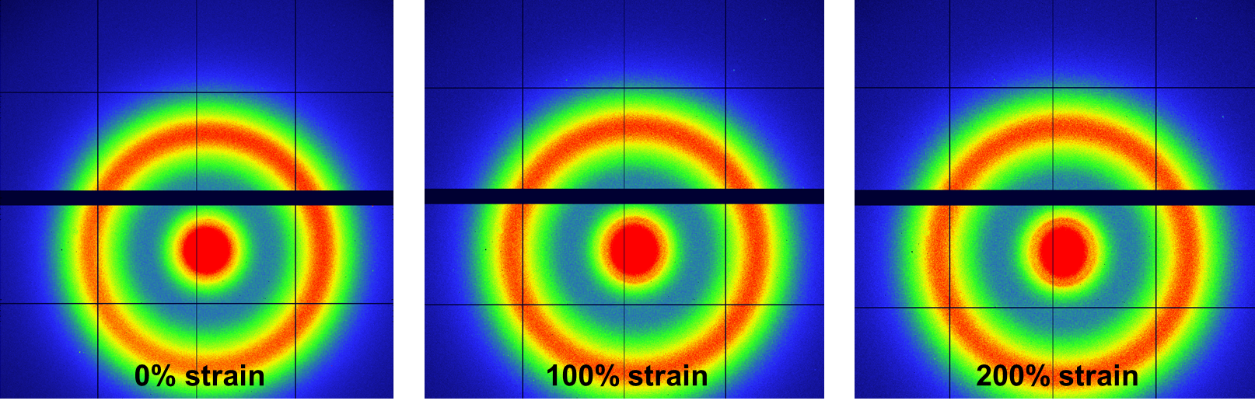


**Figure S10.** WAXD images of in-situ stretching of pure eutectogel (0%, 100%, 200%).


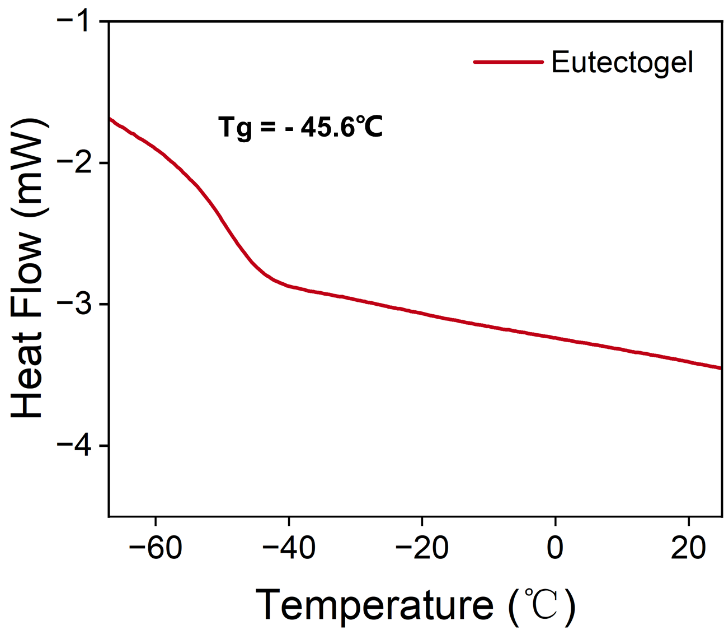


**Figure S11.** DSC curve of pure eutectogel.


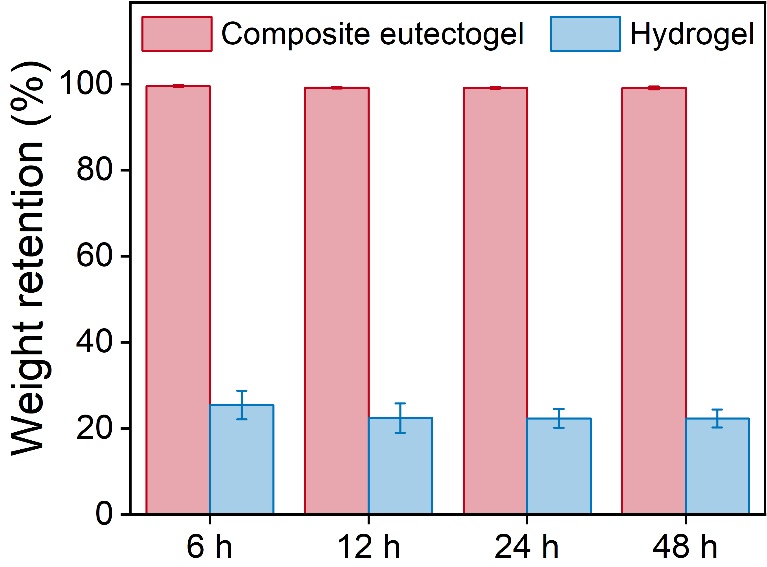


**Figure S12.** Weight retention of composite eutectogel and PVA hydrogel thermal treated at 50 °C for different times.


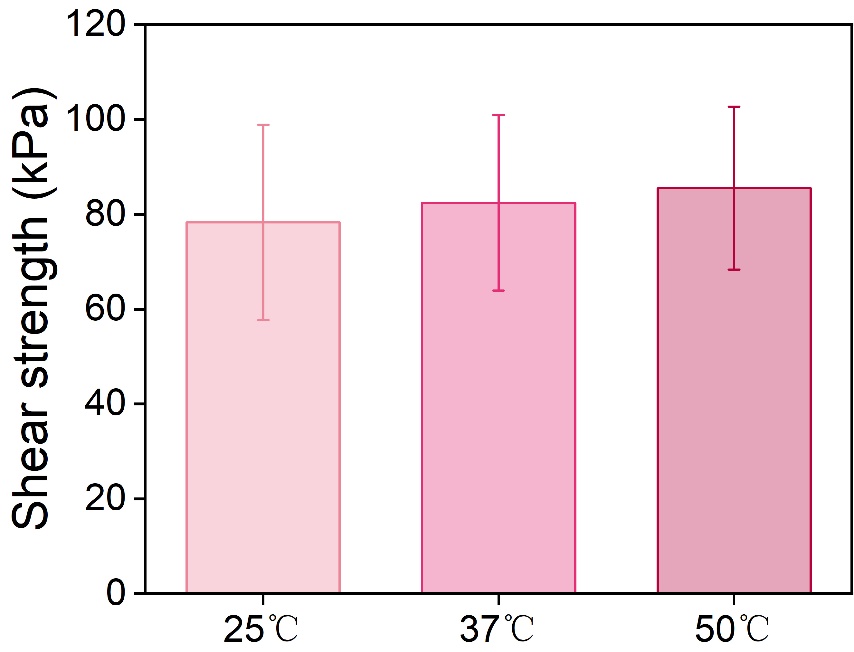


**Figure S13.** Shear strength of composite eutectogel on porcine skin at different temperatures.


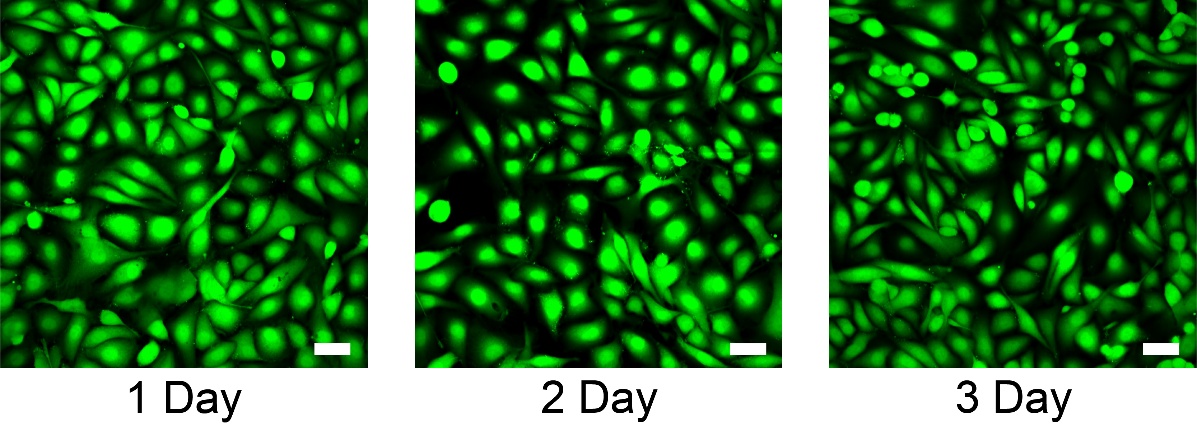


**Figure S14.** Fluorescence cell images of control group. Scale bar, 50 µm.


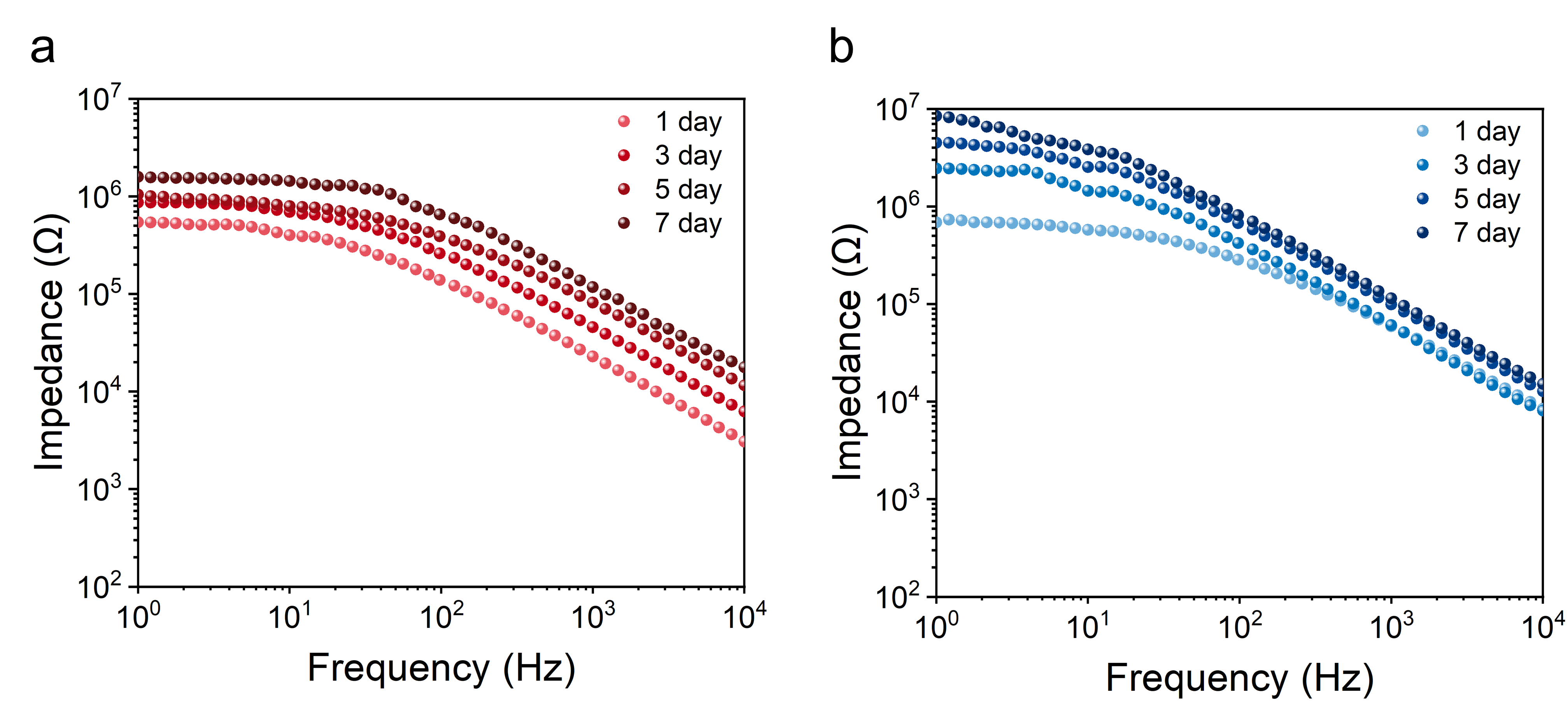


**Figure S15.** Interfacial impedance of (a) composite eutectogels and (b) commercial Ag/AgCl electrodes tested continuously for 7 days.


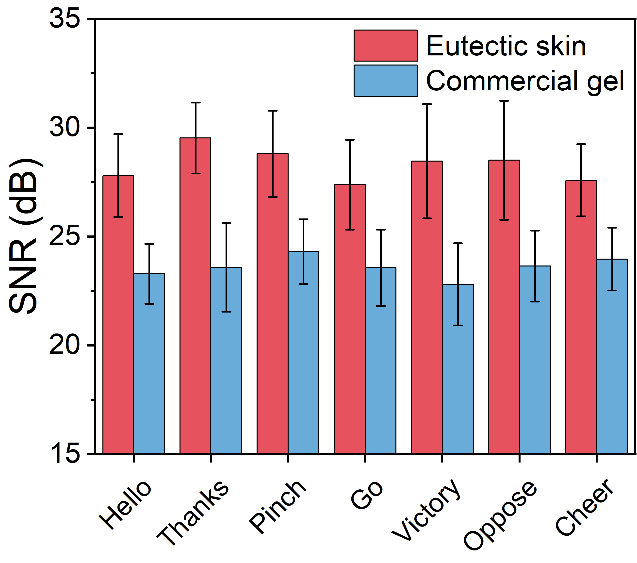


**Figure S16.** SNR values in different sign languages using eutectic skin and commercial gel elelctrodes.


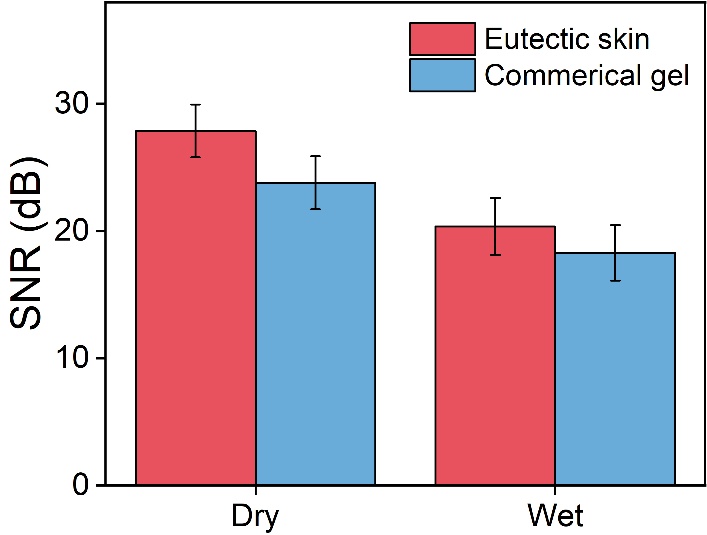


**Figure S17.** SNR values in air and underwater environments using eutectic skin and commercial gel elelctrodes.

**
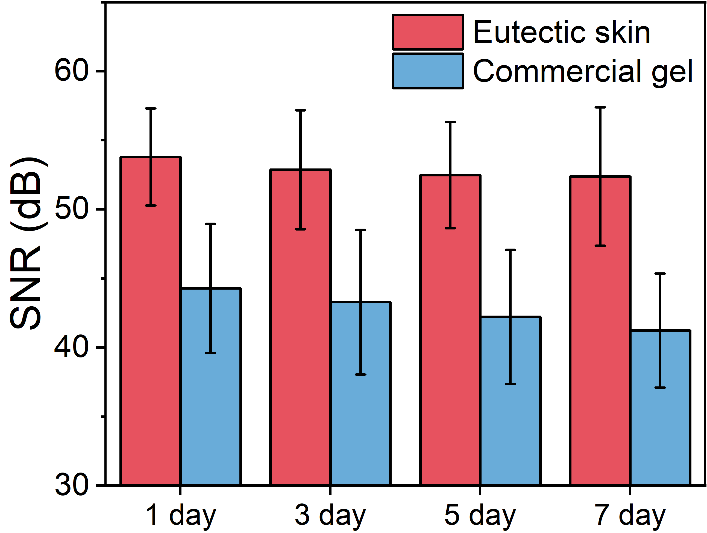
**

**Figure S18.** SNR values of ECG signals at different days using eutectic skin and commercial gel elelctrodes.

**Supplementary Tables**

**Table S1.** Comparison of the contact angles and weight swelling ratio of different eutectogel components

| EHA  (wt%) | AA  (wt%) | MMA  (wt%) | Contact angle  (°) | Weight swelling ratio for 100 days (%) |
| --- | --- | --- | --- | --- |
| 0 | 40 | 3 | 30.66±1.80 | 8.12±0.39 |
| 10 | 30 | 3 | 33.54±2.44 | 7.59±0.35 |
| 20 | 20 | 3 | 77.10±4.47 | 3.28±0.25 |
| 30 | 10 | 3 | 112.14±6.15 | 1.21±0.2 |
| 40 | 0 | 3 | 94.82±3.16 | 1.08±0.16 |

**Table S2.** Comparison of swelling ratio of eutectogel and previously reported eutectogels and hydrogels

| Cat. | Materials | Weight swelling ratio  (%) | Ref. |
| --- | --- | --- | --- |
| Eutectogel | **Composite eutectogel** | **1.08** | **This work** |
|  | PAB-DES | 80 | [1] |
|  | TFEA-HEMA-AA | 28 | [2] |
|  | BA-AA | 17-120 | [3] |
|  | BA-EHA-PEGDA | 62 | [4] |
|  | PSDIC-gel | 60-300 | [5] |
|  | BA-IBA | 85-100 | [6] |
| Hydrogel | UVR-B-DES | 180-220 | [7] |
|  | HPAE | 112 | [8] |
|  | RBV-PVA | 170-240 | [9] |
|  | PEG | 100-270 | [10] |
|  | HA-PVA | 9 | [11] |
|  | SF/TA@PPy | 13 | [12] |
|  | PAHCT | 5.5 | [13] |
|  | P(AAm-LMA-EGD) | 20-60 | [14] |
|  | S-PM | 180 | [15] |
|  | SBMA-AA-PVA | 78-150 | [16] |

**Table S3.** Comparison of mechanical performance of eutectogel and previously reported gels and elastomers

| Materials | Modulus  (MPa) | Toughness (MJ m^-3^) | Fracture  Energy (kJ m^-2^) | Fatigue thresholds  (J m^-2^) | Ref. |
| --- | --- | --- | --- | --- | --- |
| **Composite eutectogel** | **7.66** | **39.73** | **189.3** | **50234** | **This work** |
| Stiff skeleton-reinforced hydrogel | 0.18 | 0.61 | 4.6 | 441 | [17] |
| PDMS fiber-reinforced hydrogel | 0.26 | 0.12 | 4.1 | 1290 | [18] |
| TPN hydrogel | 0.45 | 0.65 | 4.2 | N/A | [19] |
| FC-A PVA hydrogel | 8 | 55 | 84.6 | 2740 | [20] |
| Annealed PVA hydrogel | 9.5 | 13.25 | 9 | 1000 | [21] |
| HA-PVA hydrogel | 8 | 210 | 175 | 10500 | [22] |
| PVA-ferro/ferricyanide | 2 | 12.5 | 17.9 | 2500 | [23] |
| PAAm/Ca-alginate | 1.19 | 0.781 | 3.4 | 35 | [24] |
| PAMPS/PAAM hydrogel | 0.5 | 2.47 | 3.8 | 418 | [25] |
| Highly entangled hydrogel | 0.1 | 0.68 | 1.46 | 200 | [26] |
| PAA-G8 hydrogel | 0.012 | 0.69 | 0.9 | 126 | [27] |
| PAAm hydrogel | 0.048 | 0.42 | 0.6 | 64.5 | [28] |
| Ecoflex composite | 0.05 | 0.97 | 5.7 | 500 | [29] |
| PDMS composite | 1.13 | 0.56 | 10 | 160 | [30] |
| P(MEA-co-AMPS) elastomer | 1.83 | 22 | 10.8 | 614 | [31] |
| Highly entangled elastomer | 0.54 | 14.5 | 2.2 | 240 | [26] |
| PEGA-co-MMA-Li elastomer | 18 | 228 | 95 | 2682 | [32] |
| P(AAm-co-AA) ionogel | 46.5 | 44 | 24 | N/A | [33] |
| P(NaSS-co-DMAEA-Q) | 0.8 | 1.8 | 3.65 | 150 | [34] |

**Table S4.** Surface energy of different substrates

| Substrate | Surface energy (mN m^-1^) | Ref. |
| --- | --- | --- |
| Glass | 47 | [35] |
| Fe | 46 | [36] |
| Wood | 30 | [37] |
| PET | 40 | [38] |
| PTFE | 20 | [39] |
| Human skin | 21 | [40] |

**Table S5.** Comparison of adhesion performance of eutectogel and previously reported gels

| Materials | Shear strength (kPa) | Ref. |
| --- | --- | --- |
| **Composite eutectogel** | **186.4** | **This work** |
| Poly(cation-adj-p) gels | 20-60 | [41] |
| P(MATAC-co-AAm) | 10-20 | [42] |
| P(AAc-co-MEA-co-Aa-co-AU) | 5-25 | [43] |
| Tetra-PEG with NHS esters | 21 | [44] |
| PAAM hydrogel | 130 | [45] |
| PDA-PAM | 20-25 | [46] |
| PEGS-based JAP | 20-125 | [47] |
| ACTC elastomer | 20-78 | [48] |
| GAE Hydrogel | 140-170 | [49] |
| PAAS hydrogel | 22-88 | [50] |
| PVA-PAAc-N | 63.1 | [51] |
| PAA/LMA eutectogel | 20-130 | [52] |
| Ca@SP/TA | 19.3 | [53] |
| HSCP hydrogel | 0.9-1.9 | [54] |
| RHOCF hydrogel | 6-21 | [55] |
| PAA/M-HPMC | 8.3 | [56] |

**Table S6.** Comparison of SNR of eutectogel and previously reported electrodes

| Materials | SNR (dB) | Ref. |
| --- | --- | --- |
| **Composite eutectogel** | **27.9** | **This work** |
| Ag/AgCl | 23.7 | Commercial electrodes |
| PAA-Ag-SEBS | 13 | [57] |
| PGEH | 25.2 | [58] |
| PEDOT:PSS-WPU | 11 | [59] |
| AMPS-MAEDS-AAm | 21.8 | [60] |
| n-doped P(PyV)-H | 15.8 | [61] |
| GBAH | 23.9 | [62] |
| NAGA-HACC | 20 | [63] |
| PAM/IC | 27 | [64] |
| PCA-Na | 23.3 | [65] |
| ABLE | 26.8 | [66] |
| PAAm-Gelatin-SA | 25 | [67] |
| PVA-PAAC | 22 | [68] |
| NF-vOECT | 23.1 | [69] |
| Ti_3_C_2_-Mxene-CNT | 25 | [70] |
| PEG-PAA-PAMPS | 25 | [71] |
| PEDOT:PSS-PVA | 22 | [72] |
| IPNCHs | 16.4 | [73] |
| PEDOT:PSS-PDA | 24.6 | [74] |
| Pt-Lme | 23 | [75] |

**Legends for Video S1 to S6**

**Video S1.** Limiting Ball Diameter Stop Test: Composite eutectogel

**Video S2.** Limiting Ball Diameter Stop Test: Commercial adhesives

**Video S3.** Limiting Ball Diameter Stop Test: Composite eutectogel

**Video S4.** Limiting Ball Diameter Stop Test: Commercial adhesives

**Video S5.** Underwater adhesion demonstration

**Video S6.** Underwater EMG Signal Test

**Reference**

1. H. Zheng, M. Chen, Y. Sun, B. Zuo, *Chem. Eng. J.* 2022, 446, 136931.
2. Z. Zhang, A. Yao, P. Raffa, *Chem. Eng. J.* 2025, 522, 167958.
3. H. Wang, Y. Rong, G. Qin, Z. Zhao, P. Cui, X. Zhang, R. Hang, X. Yao, X. Huang, *Colloids Surf. Physicochem. Eng. Asp.* 2025, 709, 136108.
4. C. Chai, L. Ma, Y. Chu, W. Li, Y. Qian, J. Hao, *J. Colloid Interface Sci.* 2023, 638, 439.
5. J. L. De Lacalle, M. L. Picchio, A. Dominguez-Alfaro, R. R.-M. Serrano, B. Marchiori, I. D. Agua, N. Lopez-Larrea, M. Criado-Gonzalez, G. G. Malliaras, D. Mecerreyes, *ACS Mater. Lett.* 2023, 5, 3340.
6. H. Ma, M. Wang, J. Hou, X. Wang, P. Sun, F. Wang, *Adv. Mater.* 2025, 37, 2500770.
7. T. H. Vo, P. K. Lam, F.-K. Shieh, Y.-J. Sheng, H.-K. Tsao, *Chem. Mater.* 2025, 37, 9172.
8. C. Zhang, H. He, Y. Shen, F. Kang, H. Zhai, *Polymer* 2025, 325, 128244.
9. H. Zhang, Y. Dong, L. Wang, G. Wang, J. Wu, Y. Zheng, H. Yang, S. Zhu, *J. Mater. Chem.* 2011, 21, 13530.
10. H. Lin, Y. Li, R. He, W. Zheng, Y. Lai, Y. Xu, B. Zeng, C. Yuan, L. Dai, *Adv. Funct. Mater.* 2025, 35, 2507606
11. Z. Qin, X. Yu, H. Wu, J. Li, H. Lv, X. Yang, *Biomacromolecules* 2019, 20, 3399.
12. M. Hua, S. Wu, Y. Ma, Y. Zhao, Z. Chen, I. Frenkel, J. Strzalka, H. Zhou, X. Zhu, X. He, *Nature* 2021, 590, 594.
13. R. Wu, T. Zhu, Y. Ni, C. Wu, W. Wang, K. Zhao, J. Huang, Y. Lai, *Adv. Funct. Mater.* 2025, e15120.
14. L. Sun, B. Luo, Z. Sun, X. Ren, J. Xu, *Mater. Today Commun.* 2025, 46, 112605.
15. S. Zhang, F. Guo, X. Gao, M. Yang, X. Huang, D. Zhang, X. Li, Y. Zhang, Y. Shang, A. Cao, *Adv. Sci.* 2024, 11, 2405880.
16. Y. Bai, X. Li, Y. Shi, Y. Zhang, S. Xie, *Small* 2025, 21, e08493.
17. H. Yang, M. Ji, M. Yang, M. Shi, Y. Pan, Y. Zhou, H. J. Qi, Z. Suo, J. Tang, *Matter* 2021, 4, 1935.
18. C. Xiang, Z. Wang, C. Yang, X. Yao, Y. Wang, Z. Suo, *Mater. Today* 2020, 34, 7.
19. X. Liu, J. Wu, K. Qiao, G. Liu, Z. Wang, T. Lu, Z. Suo, J. Hu, *Nat. Commun.* 2022, 13, 1622.
20. X. Liang, G. Chen, S. Lin, J. Zhang, L. Wang, P. Zhang, Z. Wang, Z. Wang, Y. Lan, Q. Ge, J. Liu, *Adv. Mater.* 2021, 33, 2102011.
21. S. Lin, X. Liu, J. Liu, H. Yuk, H.-C. Loh, G. A. Parada, C. Settens, J. Song, A. Masic, G. H. McKinley, X. Zhao, *Sci. Adv.* 2019, 5, ea8528.
22. M. Hua, S. Wu, Y. Ma, Y. Zhao, Z. Chen, I. Frenkel, J. Strzalka, H. Zhou, X. Zhu, X. He, *Nature* 2021, 590, 594.
23. Z. Lei, W. Gao, W. Zhu, P. Wu, *Adv. Funct. Mater.* 2022, 32, 2201021.
24. W. Zhang, J. Hu, J. Tang, Z. Wang, J. Wang, T. Lu, Z. Suo, *ACS Macro Lett.* 2019, 8, 17.
25. W. Zhang, X. Liu, J. Wang, J. Tang, J. Hu, T. Lu, Z. Suo, *Eng. Fract. Mech.* 2018, 187, 74.
26. J. Kim, G. Zhang, M. Shi, Z. Suo, *Science* 2021, 374, 6564
27. H. Lei, L. Dong, Y. Li, J. Zhang, H. Chen, J. Wu, Y. Zhang, Q. Fan, B. Xue, M. Qin, B. Chen, Y. Cao, W. Wang, *Nat. Commun.* 2020, 11, 4032.
28. E. Zhang, R. Bai, X. P. Morelle, Z. Suo, *Soft Matter* 2018, 14, 3563.
29. C. Li, H. Yang, Z. Suo, J. Tang, *J. Mech. Phys. Solids* 2020, 134, 103751.
30. Z. Wang, C. Xiang, X. Yao, P. Le Floch, J. Mendez, Z. Suo, *Proc. Natl. Acad. Sci.* 2019, 116, 5967.
31. Y. Zheng, R. Kiyama, T. Matsuda, K. Cui, X. Li, W. Cui, Y. Guo, T. Nakajima, T. Kurokawa, J. P. Gong, Chem. Mater. 2021, 33, 3321.
32. M. Li, L. Chen, Y. Li, X. Dai, Z. Jin, Y. Zhang, W. Feng, L.-T. Yan, Y. Cao, C. Wang, *Nat. Commun.* 2022, 13, 2279.
33. M. Wang, P. Zhang, M. Shamsi, J. L. Thelen, W. Qian, V. K. Truong, J. Ma, J. Hu, M. D. Dickey, *Nat. Mater.* 2022, 21, 359.
34. X. Li, K. Cui, T. Kurokawa, Y. N. Ye, T. L. Sun, C. Yu, C. Creton, J. P. Gong, *Sci. Adv.* 2021, 7, eabe8210.
35. D. Li, M. Xiong, S. Wang, X. Chen, S. Wang, Q. Zeng, *Appl. Surf. Sci.* 2020, 503, 144257.
36. S. Li, L. Yang, J. Christudasjustus, N. R. Overman, B. D. Wirth, M. L. Sushko, P. Simonnin, D. K. Schreiber, F. Gao, C. Wang, *Nat. Commun.* 2024, 15, 6149.
37. X. Wang, F. Wang, Z. Yu, Y. Zhang, C. Qi, L. Du, *J. Wood Sci.* 2017, 63, 271.
38. F. Rezaei, M. D. Dickey, M. Bourham, P. J. Hauser, *Surf. Coat. Technol.* 2017, 309, 371.
39. Y. Liu, L. Zhou, T. Zheng, H. Li, D. Zhang, D. Seveno, *Appl. Surf. Sci.* 2025, 684, 161829.
40. S. A. Ranamukhaarachchi, S. Lehnert, S. L. Ranamukhaarachchi, L. Sprenger, T. Schneider, I. Mansoor, K. Rai, U. O. Häfeli, B. Stoeber, *Sci. Rep.* 2016, 6, 32074.
41. X. Zhang, Q. Fu, Y. Wang, H. Zhao, S. Hao, C. Ma, F. Xu, J. Yang, *Adv. Funct. Mater.* 2024, 34, 2307400.
42. X. Liu, Q. Zhang, G. Gao, *Chem. Eng. J.* 2020, 394, 124898.
43. H. An, M. Zhang, Z. Huang, Y. Xu, S. Ji, Z. Gu, P. Zhang, Y. Wen, *Adv. Mater.* 2024, 36, 2310164.
44. Y. Bu, L. Zhang, G. Sun, F. Sun, J. Liu, F. Yang, P. Tang, D. Wu, *Adv. Mater.* 2019, 31, 1901580.
45. F. J. Cedano-Serrano, U. Sidoli, A. Synytska, Y. Tran, D. Hourdet, C. Creton, *Macromolecules* 2019, 52, 3852.
46. K. Chen, C. Liu, J. Huang, L. Che, Y. Yuan, C. Liu, *Adv. Funct. Mater.* 2023, 33, 2303836.
47. H. Fan, J. Wang, Z. Tao, J. Huang, P. Rao, T. Kurokawa, J. P. Gong, *Nat. Commun.* 2019, 10, 5127.
48. Y. Hou, Y. Li, Y. Li, D. Li, T. Guo, X. Deng, H. Zhang, C. Xie, X. Lu, *ACS Nano* 2023, 17, 2745.
49. Q. Li, P. Zhang, C. Yang, H. Duan, W. Hong, *Extreme Mech. Lett.* 2021, 43, 101193.
50. K. Shen, Z. Lv, Y. Yang, H. Wang, J. Liu, Q. Chen, Z. Liu, M. Zhang, J. Liu, Y. Cheng, *Adv. Mater.* 2025, 37, 2414092.
51. P. Ma, W. Liang, R. Huang, B. Zheng, K. Feng, W. He, Z. Huang, H. Shen, H. Wang, D. Wu, *Adv. Mater.* 2024, 36, 2305400.
52. G. Zeng, S. Chen, Y. Zhou, G. Li, G. Li, J. Zhu, C. Xu, Y. Xin, *ACS Appl. Mater. Interfaces* 2025, 17, 38532.
53. J. p. Gong, Y. Katsuyama, T. Kurokawa, Y. Osada, *Adv. Mater.* 2003, 15, 1155.
54. M. Xiao, Y. Luo, H. Chen, H. Wang, D. Yao, D. Wang, Y. Pan, B. Zha, Q. Yu, R. Xie, B.-R. Yang, K. Tao, J. Fu, F. Huo, J. Wu, *Adv. Funct. Mater.* 2025, 35, e22153.
55. X. Guo, L. Zhang, H. Zhuo, C. Chen, H. Yang, T. Li, H. Qi, W. Zhai, *Nat. Commun.* 2025, 16, 9454.
56. Q. Chen, S. Li, K. Li, W. Zhao, C. Zhao, *Adv. Sci.* 2024, 11, 2306018.
57. Y. Peng, J. Song, Y. Zhang, H. Liu, J. Dong, H. Huang, M. Weng, Y. Huang, *Adv. Mater.* 2025, 37, e08041.
58. K. Zheng, C. Zheng, L. Zhu, B. Yang, X. Jin, S. Wang, Z. Song, J. Liu, Y. Xiong, F. Tian, R. Cai, B. Hu, *Nano-Micro Lett.* 2025, 17, 281.
59. V. Mottini, L. Xing, Y. Cai, C. Meilinger, S. Inamdar, X. C. Chen, B. T. Safa, J. Wang, Y. Xing, J. Darbonne, Z. Tang, Y. Zhang, C. H. Contag, R. Yang, M. Zhang, J. Li, *Device* 2026, 4, 100987.
60. C. He, J. Zhang, H. Wang, D. Wu, H. Zheng, Z. Guo, J. Xie, W. Zhu, M. Xie, J. Zhong, Y. Liu, Z. Li, G. Lin, Z. Peng, *Adv. Funct. Mater.* 2026, 36, e09372.
61. P. Li, W. Sun, J. Li, J.-P. Chen, X. Wang, Z. Mei, G. Jin, Y. Lei, R. Xin, M. Yang, J. Xu, X. Pan, C. Song, X.Y. Deng, X. Lei, K. Liu, X. Wang, Y. Zheng, J. Zhu, S. Lv, Z. Zhang, X. Dai, T. Lei, *Science* 2024, 384, 557.
62. L. Li, Y. Sun, J. Ding, C. Wang, Y. Xiang, B. Guo, L. Liang, X. Lu, H. Zhang, F. Yao, J. Li, *Chem. Eng. J.* 2025, 519, 164901.
63. D. Wang, H. Xue, L. Xia, Z. Li, Y. Zhao, X. Fan, K. Sun, H. Wang, T. Hamalainen, C. Zhang, F. Cong, Y. Li, F. Song, J. Lin, *Microsyst. Nanoeng.* 2025, 11, 105.
64. M. Lu, L. Shen, H. Su, B. Li, L. Wang, W. W. Yu, J. *Colloid Interface Sci.* 2025, 684, 272.
65. M. Zhang, M. Hao, G. Ren, Y. Zhao, C. Lv, Y. Xia, W. Wang, W. Chen, Y. Chen, L. Li, Q. Lu, T. Zhang, *InfoMat* 2025, 7, e70066.
66. J. Shi, S. Kim, P. Li, F. Dong, C. Yang, B. Nam, C. Han, E. Eig, L. L. Shi, S. Niu, J. Yue, B. Tian, *Science* 2024, 384, 6699
67. X. Li, Y. Sun, S. Wang, G. Tian, T. Yang, L. Huang, Y. Ao, B. Lan, J. Zhang, T. Xu, Y. Liu, L. Jin, W. Yang, W. Deng, *Chem. Eng. J.* 2024, 498, 155195.
68. G. Yang, Z. Lan, H. Gong, J. Wen, B. Pang, Y. Qiu, Y. Zhang, W. Guo, T. Bu, B. Xie, H. Wu, *Adv. Funct. Mater.* 2025, 35, 2417841.
69. X. Li, Y. Zhong, Y. Wang, H. Jiang, R. Wang, W. Chen, X. Wang, R. Jia, Q. Liang, Y. Zhu, M. Zhu, Y. Sun, H. Sun, H. Jiang, G. Wang, *Adv. Mater.* 2026, 38, e11945.
70. L. Cheng, A. Guo, J. Li, M. Li, Q. Lei, W. Xu, X. Guo, J. Zhang, Sci. *China Mater.* 2024, 67, 2977.
71. Y. Li, Y. Gu, S. Qian, Y. Pang, A. Yu, S. Zheng, W. Xia, Y. Liao, B. Liu, S. Liu, Q. Zhao, *Sci. China Inf. Sci.* 2025, 68, 129402.
72. Y. Li, Y. Gu, S. Qian, S. Zheng, Y. Pang, L. Wang, B. Liu, S. Liu, Q. Zhao, *Nano Res.* 2024, 17, 5479.
73. T. Wang, J. Liu, Y. Zhao, Y. Lu, *Bioact. Mater.* 2025, 52, 300.
74. Z. Xu, H. Guo, J. Cai, F. Wang, J. Li, Q. Tang, J. Yu, B. Ding, Z. Li, *Nano Energy* 2026, 148, 111703.
75. M. Kim, E. J. Jeon, W. G. Chung, H. Kim, E. Kim, S. Lee, J.-H. Lee, S.-W. Cho, J.-U. Park, *Adv. Mater.* 2025, 37, 2419250.
